# Risk of Diabetic Ketoacidosis Associated with Sodium Glucose Cotransporter-2 Inhibitors: A Network Meta-Analysis and Meta-Regression

**DOI:** 10.3390/jcm13061748

**Published:** 2024-03-18

**Authors:** Kannan Sridharan, Gowri Sivaramakrishnan

**Affiliations:** 1 Department of Pharmacology & Therapeutics, College of Medicine & Medical Sciences, Arabian Gulf University, Manama, Bahrain; 2 Dental Post Graduate Training Department, PHCC—Primary Health Care Centers, Manama, Bahrain; gowri.sivaramakrishnan@gmail.com

**Keywords:** canagliflozin, dapagliflozin, empagliflozin, genital infections, DKA, SGLT2i

## Abstract

**Background:** Sodium glucose cotransporter-2 inhibitors (SGLT2is) represent an emerging class of drugs with diverse indications. Despite their therapeutic potential, concerns regarding safety, particularly diabetic ketoacidosis (DKA), remain contentious, with uncertainty regarding differences among various SGLT2is. This study aimed to conduct a network meta-analysis and meta-regression to evaluate the risk of SGLT2i-induced DKA and associated factors. **Methods:** We systematically searched electronic databases for randomized clinical trials assessing SGLT2is across indications, reporting incidences of DKA. Mixed treatment comparison pooled estimates (MTCPEs) were calculated, and odds ratios (OR) with 95% confidence intervals (95% CI) served as effect estimates. We analyzed differences across dose categories (low, medium, and high) and conducted a meta-regression analysis to identify risk factors. The strength of evidence for key comparisons was determined. **Results:** Our analysis included 73 articles encompassing 85,997 participants assessing the risk of DKA. SGLT2is were associated with a heightened risk of DKA compared to placebo/control interventions (OR: 1.83; 95% CI: 1.35, 2.46), a finding confirmed by bootstrap analysis. Among SGLT2is, dapagliflozin (OR: 1.9; 95% CI: 1.17, 3.08), sotagliflozin (OR: 1.93; 95% CI: 1.14, 3.25), canagliflozin (OR: 1.11; 95% CI: 1.11, 12.45), and ertugliflozin (OR: 3.92; 95% CI: 1.04, 14.77) exhibited increased DKA risk. No significant differences were observed among specific SGLT2is. Sub-group analyses revealed a high risk of DKA with low (OR: 1.98; 95% CI: 1.3, 2.95) and high doses (OR: 2.4; 95% CI: 1.7, 3.3), type 1 diabetes (OR: 3.6; 95% CI: 1.6, 8.1), type 2 diabetes (OR: 1.6; 95% CI: 1.3, 2.4), as well as a diabetes duration exceeding 10 years (OR: 3.4; 95% CI: 1.1, 10.8). The evidence of certainty for most comparisons was moderate. **Conclusions:** SGLT2 inhibitors (SGLT2is) have been found to elevate the risk of DKA. The key factors that significantly predict the likelihood of DKA include the presence of diabetes (whether T1D or T2D) and the duration of diabetes. Based on these findings, standard treatment guidelines should advise taking specific precautions against DKA in patients identified as high-risk.

## 1. Introduction

Sodium glucose cotransporter-2 inhibitors (SGLT2is) have garnered approval for treating type 2 diabetes (T2D), heart failure, and chronic kidney disease [1]. Moreover, SGLT2is find utility as off-label medications in conditions like non-alcoholic fatty liver disease and neurodegenerative disorders [2]. Despite regulatory authorities expressing reservations about their efficacy claims and suggesting that the anti-diabetic effect plateaus after several months [3], several guidelines continue to advocate for the use of SGLT2is. In addition to their anti-hyperglycemic action, SGLT2is demonstrate several pleiotropic mechanisms, including reducing systolic blood pressure and exerting anti-inflammatory and antifibrotic effects [4]. Despite these advantages, concerns about the safety of SGLT2is have been raised, encompassing issues such as hypoglycemia, lower limb amputation, acute kidney injury, urinary tract infections (UTIs), and genital infections [5].

SGLT2is function by decreasing glucose reabsorption from the proximal convoluted tubule and increasing urinary glucose excretion [6]. Consequently, this leads to reduced carbohydrate usage and prompts a shift toward the utilization of fatty acids, resulting in ketogenesis, a process observed as diabetic ketoacidosis (DKA) in diabetic patients [7]. Studies in rats have shown that SGLT2is can increase plasma concentrations of catecholamines and cortisol due to hypovolemia, leading to insulinopenia and subsequently augmenting hepatic ketogenesis [8]. In 2015, the USFDA updated the label for SGLT2is to include information about the risk of DKA [9]. However, conflicting results have emerged regarding the risk of DKA associated with SGLT2is. Donnan et al., in a meta-analysis of 26 randomized clinical trials (RCTs), found no significant risk with SGLT2is (relative risk: 0.66, 95% confidence intervals: 0.3, 1.45) [10]. Conversely, Liu et al., in a study involving 39 RCTs, observed an increased risk of DKA with an odds ratio of 2.13 [11]. Another meta-analysis of seven RCTs revealed a 2.5-fold higher risk of DKA [12]. A meta-analysis of 16 real-world studies showed that SGLT2is increased the risk of DKA by 33% in patients with T2D [13].

Existing reviews and meta-analyses on this subject have several limitations: there are few clinical trials directly comparing SGLT2is, making it unclear whether differences exist among various SGLT2is; many studies did not evaluate the strength of evidence, making decision-making challenging for clinicians; evaluations were often limited to specific indications such as T2D, heart failure, and chronic kidney disease; and risk factors were not consistently identified using meta-regression analysis.

Network meta-analysis is a statistical technique used to estimate treatment differences in outcomes between interventions when head-to-head clinical trials are lacking, using a common comparator [14]. In this study, we conducted a network meta-analysis with meta-regression to assess the risk of DKA with SGLT2is, investigate intra-class differences, and identify associated predictive factors.

## 2. Materials and Methods

### 2.1. Search Strategy

The protocol for this systematic review is available at https://osf.io/5fwyk (accessed on 9 December 2023). We conducted searches across several databases, including PubMed, Cochrane Central, and Google Scholar. The search strategy employed in this study is summarized in Electronic Supplementary Table S1, with the latest search conducted on 9 December 2023. No restrictions were imposed on the publication year or language. Conference proceedings were excluded from consideration. Additionally, references cited in eligible studies were cross-checked for relevance to this meta-analysis. This study adheres to the reporting guidelines outlined in the Preferred Reporting Items for Systematic Reviews and Meta-Analyses (PRISMA) statement, with specific adherence to the network meta-analysis extension [15].

### 2.2. Eligibility Criteria

We included only randomized clinical trials that met the following criteria:

Population: Adults/children with disorders for which SGLT2i was administered.

Intervention: SGLT2i: bexagliflozin, empagliflozin, dapagliflozin, canagliflozin, enavogliflozin, ertugliflozin, henagliflozin, ipragliflozin, licogliflozin, luseogliflozin, remogliflozin, sotagliflozin, and tofogliflozin.

Control: Placebo/standard of care/any of the above SGLT2i.

Outcome: Diabetic ketoacidosis. We excluded articles that have just stated ‘ketonemia’ or ‘ketosis’ or ‘ketoacidosis’.

### 2.3. Study Procedure

Each author conducted an independent search and extracted the following details: trial identification, year, diagnosis of study participants (including type 1 diabetes (T1D), type 2 diabetes (T2D), heart failure (HF), chronic kidney disease (CKD), non-alcoholic fatty liver disease (NAFLD), acute myocardial infarction (AMI), prediabetes, and obesity), demographics of the study participants (such as age, gender distribution, baseline HbA1c, baseline estimated glomerular filtration rate (eGFR), body mass index (BMI), and duration of diabetes), and drug-related specifics (name, dose, frequency, and duration), along with outcomes. Any discrepancies in data retrieval were resolved through discussion. The Cochrane risk of bias tool [16], encompassing aspects like generation of random sequence, concealment of allocation, blinding of participants, study personnel, and outcome assessment, incomplete outcome reporting, and selective reporting of outcomes, was utilized for assessing bias risk. Publication bias for SGLT2i versus placebo/standard of care was evaluated using a funnel plot and the Begg and Mazumdar test [17].

Direct and mixed treatment comparison pooled estimates were generated using the random-effects model. Direct estimates relied on data from head-to-head clinical trials, while mixed treatment comparison pooled estimates were derived from both direct and indirect estimates. Odds ratios (95% confidence intervals) (OR 95% CI) were employed as effect estimates. Inconsistency between direct and indirect pooled estimates was evaluated using H statistics, classified as mild (<3), modest (3–6), or large (>6) [18]. Mixed comparison pooled estimates were estimated using MetaXL© (version 5.3, The Netherlands) and OpenMEE (version 1.0, USA) [19]. Bootstrap meta-analysis was carried out using the DerSimonian-Laird random effects model with 1000 replicates. A leave-one-out meta-analysis assessed the impact of excluding data from each study. Confirmation of pooled estimates for the risk of DKA with overall SGLT2is was achieved through a bootstrap meta-analysis with 1000 iterations. Grading of pooled estimates was conducted using the GRADE working group approach [20]. Subgroup analyses by dose categories (see Electronic Supplementary Table S2) and indications (T1D, T2D, and non-diabetic) were performed.

Meta-regression analysis was conducted, incorporating covariates such as age groups (<19, 20 to <40, ≥40 to <65, and ≥65 years), HbA1c levels (<5.7, 5.7–6.4, ≥6.5 to <7, and ≥7%), BMI (<25, ≥25 to <30, and ≥30 kg/m^2^), eGFR (<60, ≥60 to <90, and ≥90 mL/min/1.73 m^2^), indications for SGLT2i (T1D, T2D, and non-diabetes), duration of treatment with SGLT2i (<6 and ≥6 months), and trial design masking (double-blind, single-blind, and open label). Meta-regression utilized the maximum likelihood random-effects method, with the natural logarithm of odds (Ln(odds)) converted to OR with a 95% CI for each covariate. Meta-regression, Bootstrap meta-analysis, and leave-one-out sensitivity meta-analysis were conducted using OpenMEE [21].

## 3. Results

### 3.1. Search Results

**Figure 1:**
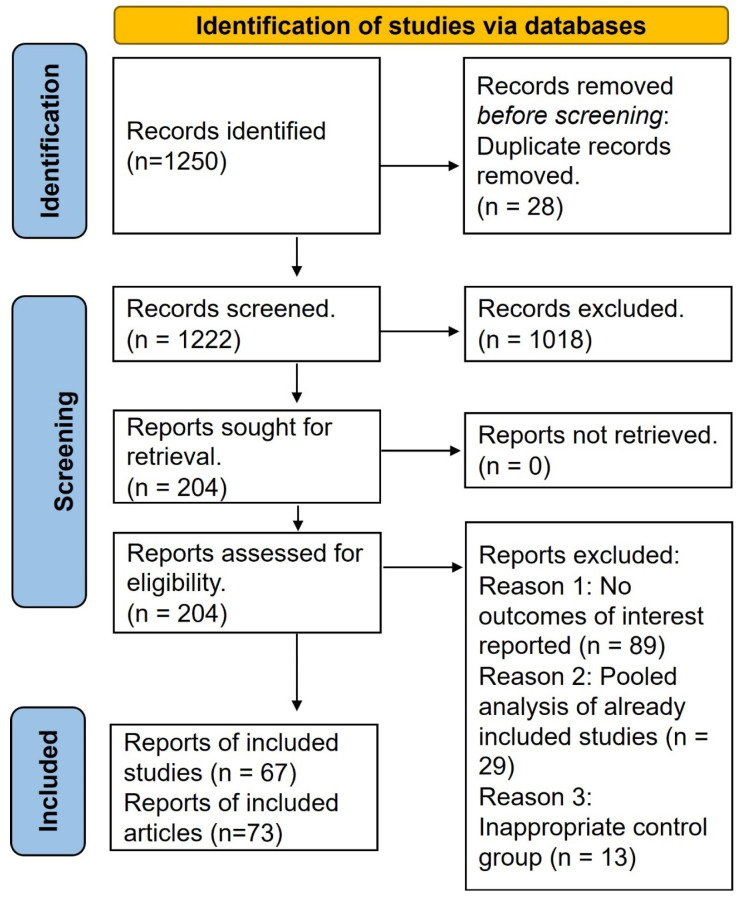
PRISMA flow chart. Seventy-three articles were included from a total of 1250 articles.

**Figure 2:**
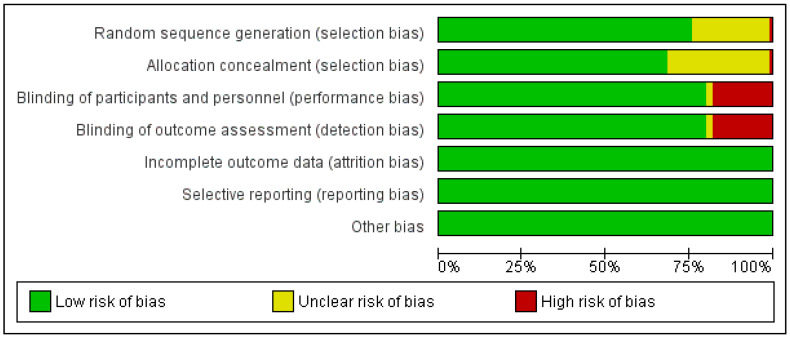
Summary of risk of bias of included studies. The green bar indicates low risk; yellow bar represents unclear risk; and red bar indicates high risk of bias. Most of the included studies had low risk of bias in all the domains.

The search strategy yielded a total of 1250 articles, ultimately resulting in the inclusion of 73 studies (67 unique studies) (Figure 1). The Electronic Supplementary Table S3 provides key characteristics of the included studies. Predominantly, the studies were focused on T2D (*n* = 30), followed by T1D (*n* = 13), T2D/CKD (*n* = 6), HF (*n* = 5), HF/T2D (*n* = 4), CKD (*n* = 3), obesity (*n* = 2), T2D/NAFLD (*n* = 1), pre-diabetes (*n* = 1), AMI (*n* = 1), and one study encompassed heart failure with or without diabetes. Most of the studies exhibited a low risk of bias across all domains (Figure 2).

### 3.2. Pooled Estimates for the Risk of DKA

**Figure 3:**
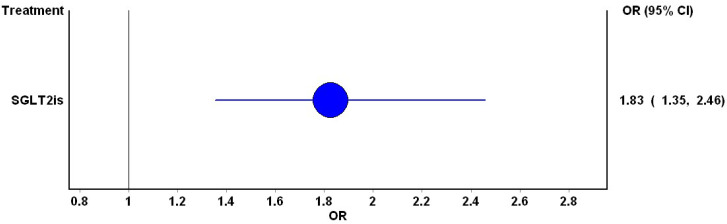
Forest plot for the mixed comparison pooled estimates for the risk of DKA with SGLTis. The blue circle represents the point estimate while the blue line represents the 95% confidence interval for the point estimate. The vertical central black line is the line of no difference.

A total of 67 studies, comprising 73 articles and involving 85,997 participants, were included in the comparison between SGLT2i and placebo/standard of care (Table 1). The overall mixed treatment comparison pooled estimates indicated an elevated risk of DKA associated with SGLT2i (OR: 1.83; 95% CI: 1.35, 2.46) (Figure 3). Notably, no significant inconsistencies were observed (H value = 1).

**Figure 4:**
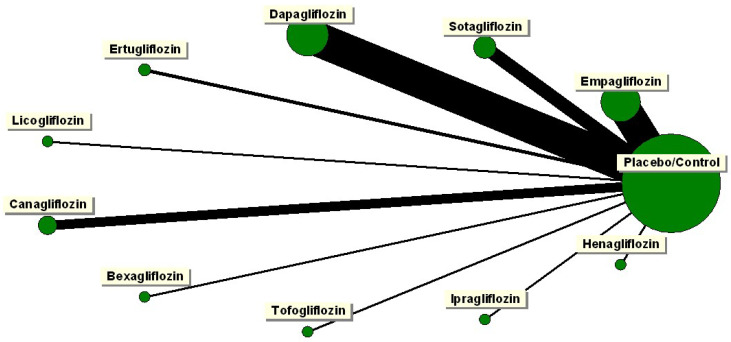
Network plot of SGLTi for the risk of DKA. The green circles represent the number of studies for each intervention and the black lines represent the number of studies comparing the respective interventions.

**Figure 5:**
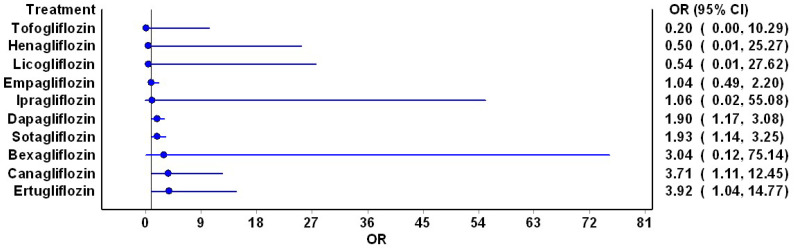
Forest plot for the risk of DKA with the mixed treatment comparison pooled estimates for SGLT2i compared to placebo/standard of care. The blue circles represent the point estimate while the blue lines represent 95% confidence intervals for the point estimates. The vertical central black line is the line of no difference.

The network plot illustrating individual SGLT2is’ risk of DKA is presented in Figure 4. Among these, dapagliflozin boasted the highest number of studies, closely followed by empagliflozin and sotagliflozin. Mixed treatment comparison pooled estimates unveiled a significantly increased risk of DKA with dapagliflozin (OR: 1.9; 95% CI: 1.17, 3.08), sotagliflozin (OR: 1.93; 95% CI: 1.14, 3.25), canagliflozin (OR: 1.11; 95% CI: 1.11, 12.45), and ertugliflozin (OR: 3.92; 95% CI: 1.04, 14.77) (Figure 5). However, mixed treatment comparison pooled estimates did not reveal any significantly different risk of DKA among SGLT2is (Table 2). The results of direct comparison pooled estimates were consistent with the mixed treatment comparison pooled estimates when compared to placebo (Table 3). However, due to the paucity of studies, no other direct comparison pooled estimates could be estimated. Notably, no significant inconsistencies were observed (H value ranged between 1 and 1.2).

#### 3.2.1. Sub-Group Analyses

**Figure 6:**
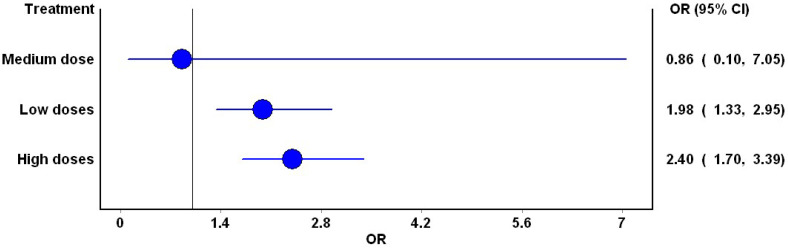
Forest plot depicting the risk of DKA with various dose categories. The blue circle represents the point estimate, while the blue line represents the 95% confidence interval for the point estimate. The vertical central black line is the line of no difference.

Most studies compared high doses (*n* = 45; 45,370 participants), with low doses following closely behind (*n* = 35; 31,924 participants) (Table 1). Mixed treatment comparison pooled estimates indicated a significantly increased risk of DKA with both low (OR: 1.98; 95% CI: 1.3, 2.95) and high doses (OR: 2.4; 95% CI: 1.7, 3.3) (Figure 6).

Regarding the sub-group analysis based on indications, the risk of DKA was highest amongst T1D (OR: 3.6; 95% CI: 1.6, 8.1), followed by T2D (OR: 1.6; 95% CI: 1.3, 2.4), and not for non-diabetic purposes (OR: 1.5; 95% CI: 0.7, 3.3) (Electronic Supplementary Figure S1).

#### 3.2.2. Bootstrap Meta-Analysis

**Figure 7:**
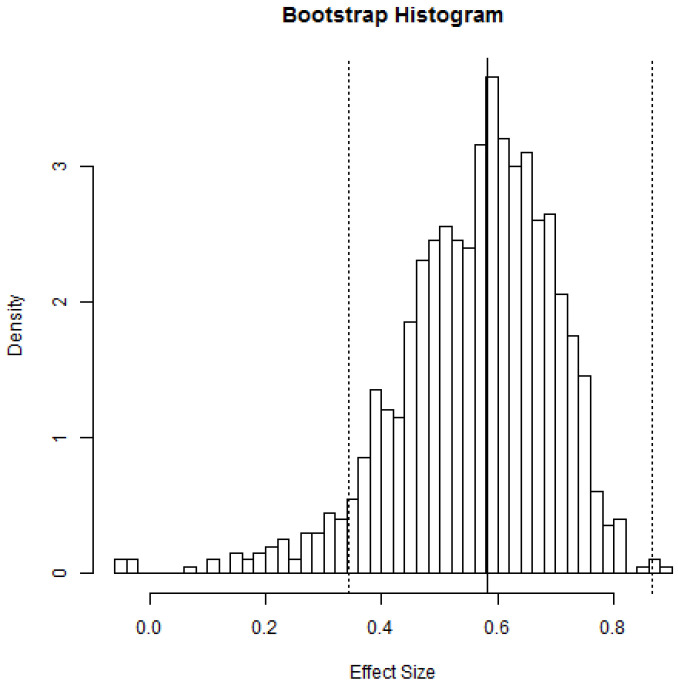
Distribution of pooled estimates following bootstrap meta-analysis. The histogram represents the distributions of Lnodds following 1000 iterations. The vertical dashed lines represent the 95% confidence intervals, and the solid vertical line indicates the point estimate. The effect sizes are mentioned in Ln (odds).

The distributions of the pooled estimates following 1000 iterations are depicted in Figure 7, and the mean pooled OR of 1.8 [1.4, 2.4] was observed.

#### 3.2.3. Meta-Regression Analyses

The pooled estimates for the covariates, as determined through meta-regression analyses, are outlined in Table 4. The sole significant predictor was the duration of diabetes, revealing an elevated risk of DKA in individuals with a diabetes duration of 10 years or more.

#### 3.2.4. Publication Bias

Funnel plot analyses did not detect any evidence of publication bias for SGLT2is, dapagliflozin, sotagliflozin, canagliflozin, or for low and high doses of SGLT2is (as illustrated in Electronic Supplementary Figures S2–S7). Additionally, Begg and Mazumdar tests did not yield significant results for any of these comparisons (*p* > 0.05).

#### 3.2.5. Leave-One-Out Sensitivity Analysis

A leave-one-out sensitivity analysis was performed by excluding data from each study, and no significant impact was observed on the overall pooled estimates for DKA (Electronic Supplementary Figure S8).

#### 3.2.6. GRADE Analysis

The strengths of evidence for key comparisons are listed in Table 5, and a moderate strength was observed for most of the estimates, mainly limited by the high risk of bias.

## 4. Discussion

### 4.1. Key Findings

Seventy-three articles were included in the evaluation of DKA risk in the present meta-analysis. It was observed that SGLT2 inhibitors (SGLT2is) exhibited an elevated risk of DKA in comparison to the placebo/control interventions. The results were confirmed through bootstrap analysis. Among the SGLT2is assessed, dapagliflozin, sotagliflozin, canagliflozin, and ertugliflozin were identified as having an increased risk of DKA. Notably, no significant differences were noted among the various SGLT2is investigated. The risk of DKA was found to be higher in both T1D and T2D populations, with no significant associations observed for other indications. Furthermore, both low and high doses of SGLT2is were associated with an elevated risk of DKA, as were longer durations of diabetes exceeding 10 years. It is important to note that a moderate level of certainty in the evidence was observed for most of the comparisons.

### 4.2. Comparison with the Existing Studies

A recent network meta-analysis, which included 36 clinical trials with 52,264 participants [22], has been the only network meta-analysis published recently on this topic. However, it focused exclusively on patients with T2D, and the overall analysis revealed no significant risk of DKA with SGLT2i. However, the authors of that study found that SGLT2is ranked third in the DKA risk following glucagon-like peptide-1 receptor agonists and dipeptidyl peptidase-4 inhibitors. Further, canagliflozin was observed to have a higher incidence of DKA in the sub-group analysis based on doses in that study. It is important to note, though, that while DKA is more prevalent in individuals with Type 1 Diabetes (T1D) than in those with T2D, it can also occur in non-diabetic conditions, such as starvation, alcoholism, pregnancy, lactation, and hyperthyroidism [23]. In our study, we found that the risk of DKA is greatest in T1D, followed by T2D, and least likely in other conditions. Despite this risk, the rarity of DKA occurrences when using SGLT2 inhibitors suggests that the benefits of this class of drugs may outweigh the risks. Our observations indicate that SGLT2 inhibitors nearly double the risk of DKA. Furthermore, both low and high doses of SGLT2 inhibitors are linked to an increased risk of DKA. Therefore, it may be wise to consider alternative anti-diabetic medications if DKA is induced by SGLT-2 inhibitors. Additionally, the Medical and Health Regulatory Authority of the United Kingdom has recently warned of an increased risk of DKA when using glucagon-like peptide drugs in combination with insulin [24]. Amongst the SGLT2is, we did not observe any increased risk with bexagliflozin, empagliflozin, henagliflozin, ipragliflozin, licogliflozin, and tofogliflozin. We could not decipher any plausible reason for this difference. However, whether this difference is clinically relevant needs to be explored in future studies.

Diabetic ketoacidosis (DKA) is a potentially life-threatening condition, making it crucial to inform patients about this significant risk, despite its rare occurrence. This warning is included in the medication guide by the US Food and Drug Administration (FDA), and patients are advised to consult healthcare professionals if they experience any early warning signs [25]. In hospital settings, the presence of a dedicated clinical pharmacist, along with vigilant monitoring of the patient’s clinical status and laboratory results, has been associated with no reported cases of DKA among patients treated with empagliflozin [26]. Meanwhile, at retail pharmacy outlets, it is vital for community pharmacists to educate diabetic patients about the risks of DKA associated with SGLT2 inhibitors. This education should particularly emphasize trigger factors, such as fasting, the consumption of low-carbohydrate diets, avoiding dehydration, and the importance of early detection of DKA symptoms.

### 4.3. Strengths and Limitations

This meta-analysis represents the most comprehensive evaluation to date of the risk of diabetic ketoacidosis (DKA) associated with SGLT2 inhibitors (SGLT2is), including the largest participant cohort ever analyzed for this purpose. Through pooled estimates of SGLT2is, our analysis has provided insights that might otherwise take decades to emerge from clinical trials. Additionally, we employed meta-regression techniques to identify risk factors. Our GRADE analysis indicates a moderate level of certainty in the evidence for most comparisons. However, the study faces limitations due to an insufficient number of studies on certain SGLT2is. This scarcity has made it challenging to assess publication bias and, consequently, to firmly establish the strength of the evidence.

## 5. Conclusions

SGLT2 inhibitors (SGLT2is) have been found to elevate the risk of DKA. The key factors that significantly predict the likelihood of DKA include the presence of diabetes (whether T1D or T2D) and the duration of the diabetes. Based on these findings, standard treatment guidelines should advise taking specific precautions against DKA in patients identified as high-risk.

## Figures and Tables

**Table 1:** Total number of studies/participants included for DKA.

Interventions	Comparators	Number of Studies	Number of Participants
SGLTi			
Bexagliflozin	Placebo/Standard of care	1	317
Canagliflozin		7	5720
Dapagliflozin		26	37,505
Empagliflozin		22	17,824
Ertugliflozin		2	8859
Henagliflozin		1	490
Ipragliflozin		1	66
Licogliflozin		1	95
Sotagliflozin		10	14,727
Tofogliflozin		1	394
Dose categories			
Low	Placebo/Standard of care	35	31,924
Medium		3	332
High		46	45,370
Low	High	18	9262

**Table 2:** Mixed treatment comparison pooled estimates of the individual SGLT2i for the risk of UTI.

Reference Intervention	Control Intervention										
	B	C	D	E	Er	H	I	L	S	T	P
B		0.8 [0.1, 25.3]	1.6 [0.1, 41]	2.9 [0.1, 78.6]	0.8 [0.1, 8.8]	6.1 [0.1, 968]	2.9 [0.01, 464]	5.7 [0.1, 913.8]	1.6 [0.1, 40.7]	15 [0.1, 2394]	3 [0.1, 75.1]
C			2 [0.5, 7.2]	3.6 [0.9, 14.8]	0.9 [0.2, 5.7]	7.4 [0.1, 452]	3.5 [0.1, 217]	6.9 [0.1, 428]	1.9 [0.5, 7.2]	18.3 [0.3, 1118]	3.7 [1.1, 12.4] *
D				1.8 [0.7, 4.4]	0.5 [0.1, 2]	3.8 [0.1, 198.4]	1.8 [0.03, 95.6]	3.5 [0.1, 188]	1 [0.5, 2]	9.4 [0.2, 491]	1.9 [1.2, 3.1] *
E					0.3 [0.1, 1.2]	2.1 [0.1, 113.4]	1 [0.1, 54.6]	1.9 [0.1, 107.5]	0.5 [0.2, 1.3]	5.1 [0.1, 280]	1 [0.5, 2.2]
Er						7.8 [0.1, 494]	3.7 [0.1, 237.9]	7.3 [0.1, 468]	2 [0.5, 8.5]	19.4 [0.3, 1223]	3.9 [1.1, 14.7] *
H							0.5 [0.01, 123.1]	0.9 [0.001, 243]	0.3 [0.001, 13.6]	2.5 [0.01, 636]	0.5 [0.01, 25.2]
I								2 [0.001, 525]	0.6 [0.01, 29.6]	5.2 [0.02, 1376]	1.1 [0.02, 55]
L									0.3 [0.01, 14.8]	2.6 [0.01, 691]	0.5 [0.01, 27.6]
S										9.5 [0.2, 500]	1.9 [1.1, 3.2] *
T											0.2 [0.004, 10.2]
P											

B: bexagliflozin; E: empagliflozin; D: dapagliflozin; C: canagliflozin; Er: ertugliflozin; H: henagliflozin; I: ipragliflozin; L: licogliflozin; S: sotagliflozin; T: tofogliflozin; P: placebo; *: statistically significant.

**Table 3:** Direct comparison pooled estimates of the individual SGLTi for the risk of DKA.

Reference Intervention	Control Intervention										
	B	C	D	E	Er	H	I	L	S	T	P
B		NA	NA	NA	NA	NA	NA	NA	NA	NA	3 [0.1, 75.1]
C			NA	NA	NA	NA	NA	NA	NA	NA	3.7 [1.1, 12.4] *
D				NA	NA	NA	NA	NA	NA	NA	1.9 [1.2, 3.1] *
E					NA	NA	NA	NA	NA	NA	1 [0.5, 2.2]
Er						NA	NA	NA	NA	NA	3.9 [1.1, 14.7] *
H							NA	NA	NA	NA	0.5 [0.01, 25.2]
I								NA	NA	NA	1.1 [0.02, 55]
L									NA	NA	0.5 [0.01, 27.6]
S										NA	1.9 [1.1, 3.2] *
T											0.2 [0.004, 10.2]
P											

B: bexagliflozin; E: empagliflozin; D: dapagliflozin; C: canagliflozin; Er: ertugliflozin; H: henagliflozin; I: ipragliflozin; L: licogliflozin; P: placebo/standard of care; S: sotagliflozin; T: tofogliflozin; NA: not available; *: statistically significant.

**Table 4:** Summary of results of meta-regression analyses.

Covariates		Odds Ratio (95% CI)
Age (compared <19) in years	20 to <40	0.7 [0.03, 16.5]
	≥40 to <65	3 [0.3, 30.7]
	≥65	2.3 [0.2, 23.5]
Indications (compared to non-diabetic)	T1D	2.4 [0.8, 7.5]
	T2D	1.1 [0.5, 2.6]
BMI (compared to <25) in kg/m^2^	≥25 to <30	4.8 [0.5, 50.8]
	≥30	4.1 [0.4, 41.6]
eGFR (compared to ≥90) mL/min/1.73 m^2^	≥60 to <90	2.1 [0.8, 5.6]
	<60	1.8 [0.7, 4.8]
Duration of diabetes (compared to 5 to 10) in years	≥10	3.4 [1.1, 10.8] *
HbA1c (compared to <5.7) in %	5.7 to 6.4	1 [0.03, 30]
	≥6.5 to <7	1.2 [0.05, 25.9]
	≥7	1.9 [0.1, 30.9]
Duration of treatment (compared to <6 months)	≥6 months	1.7 [0.8, 3.4]
Blinding design in clinical trials (compared to open label)	Double-blinded	1.9 [0.6, 6.2]
	Single-blinded	1 [0.02, 63.8]

*—Statistically significant.

**Table 5:** Summary of findings and grading the strength of evidence for key comparisons for the risk of DKA.

Comparisons with Placebo/Standard of Care (Except Gender-Wise Comparison)	Illustrative Comparative Risks (per 10,000) (95% Confidence Intervals)		Effect Estimates and the Quality of Evidence for Mixed Treatment Comparisons
	Assumed Risk ^1^	Corresponding Risk	
Any SGLT2i	10	18 (13 to 25)	1.83 [1.35, 2.46]; ⊕⊕⊕⊝; Moderate ^2^
Dapagliflozin	4	8 (5 to 12)	1.9 [1.17, 3.08]; ⊕⊕⊕⊝; Moderate ^2^
Sotagliflozin	37	71 (42 to 119)	1.93 [1.14, 3.25]; ⊕⊕⊕⊝; Moderate ^2^
Canagliflozin	1	1 (1 to 12)	1.11 [1.11, 12.45]; ⊕⊕⊝⊝; Low ^2,3^
Ertugliflozin	4	16 (4 to 58)	3.92 [1.04, 14.77]; ⊕⊝⊝; Very low ^2,3,4^
Low doses of SGLT2i	10	20 (13 to 29)	1.98 [1.3, 2.95]; ⊕⊕⊕⊝; Moderate ^2^
High doses of SGLT2i	8	19 (14 to 26)	2.4 [1.7, 3.3]; ⊕⊕⊕⊝; Moderate ^2^

^1^ Assumed risk was the mean control group risk across the studies. ^2^ Downgraded one level for including studies with high risk of bias. ^3^ Downgraded one level for serious limitations in the precision of the estimates. ^4^ Downgraded one level as publication bias could not be assessed/ruled out. Moderate: The authors believe that the true effect is probably close to the estimated effect. Low: Further research is very likely to have an important impact on our confidence in the estimate of effect and is likely to change the estimate. Very low: We have very little confidence in the effect estimate.

## Data Availability

The data shall be shared upon a reasonable request to the corresponding author.

## Supplementary Materials

The following supporting information can be downloaded at: https://www.mdpi.com/article/10.3390/jcm13061748/s1, Electronic Supplementary Table S1: Search strategy; Electronic Supplementary Table S2: Classification of doses of SGLT2i; Electronic Supplementary Table S3: Key characteristics of the included studies; Electronic Supplementary Figure S1: Forest plot for the sub-group analysis based on indications; Electronic Supplementary Figure S2: Funnel plot for the risk of DKA with SGLTi; Electronic Supplementary Figure S3: Funnel plot for the risk of DKA with dapagliflozin; Electronic Supplementary Figure S4: Funnel plot for the risk of DKA with sotagliflozin; Electronic Supplementary Figure S5: Funnel plot for the risk of DKA with canagliflozin; Electronic Supplementary Figure S6: Funnel plot for the risk of DKA with low doses of SGLT2i; Electronic Supplementary Figure S7: Funnel plot for the risk of DKA with high doses of SGLT2i; Electronic Supplementary Figure S8: Leave-one-out sensitivity analysis for the risk of DKA.
