# Supplementary material for: Risk of Diabetic Ketoacidosis Associated with Sodium Glucose Cotransporter-2 Inhibitors: A Network Meta-Analysis and Meta-Regression
Source: J Clin Med. 2024 Mar 18;13(6):1748. doi: 10.3390/jcm13061748 (PMC10971681; doi:10.3390/jcm13061748)
Supplement: Supplementary file 1 [file jcm-13-01748-s001.zip › jcm-2923976-supplementary.pdf]

Electronic Supplementary Table S1. Search strategy.

PubMed

((SGLT-2 inhibitor[Title/Abstract] OR sodium glucose transporter[Title/Abstract] OR canagliflozin[Title/Abstract] OR dapagliflozin[Title/Abstract] OR empagliflozin[Title/Abstract] OR ertugliflozin[Title/Abstract] OR sotagliflozin[Title/Abstract] OR bexagliflozin[Title/Abstract]) AND (random[Title/Abstract] OR randomized[Title/Abstract] OR randomised[Title/Abstract])) NOT (Review[Publication Type] OR Meta-analysis[Publication Type]).

Cochrane CENTRAL

**ID**	**Search**
#1	("SGLT-2"):ti,ab,kw (Word variations have been searched)
#2	("sodium glucose co-transporter 2"):ti,ab,kw (Word variations have been searched)
#3	(canagliflozin):ti,ab,kw (Word variations have been searched)
#4	(dapagliflozin):ti,ab,kw (Word variations have been searched)
#5	(empagliflozin):ti,ab,kw (Word variations have been searched)
#6	(ertugliflozin):ti,ab,kw (Word variations have been searched)
#7	(sotagliflozin):ti,ab,kw (Word variations have been searched)
#8	(bexagliflozin):ti,ab,kw (Word variations have been searched)
#9	#1 OR #2 OR #3 OR #4 OR #5 OR #6 OR #7 OR #8
#10	("randomized clinical trial"):ti,ab,kw (Word variations have been searched)
#11	#9 AND #10

Electronic Supplementary Table S2. Classification of doses of SGLT2i.

SGLT2i	Low doses (mg/day)	Medium doses (mg/day)	High doses (mg/day)
Bexagliflozin	≤5	>5 to <20	≥20
Canagliflozin	≤100	>100 to <300	≥300
Dapagliflozin	≤5	>5 to <10	≥10
Empagliflozin	≤10	>10 to <25	≥25
Ertugliflozin	≤5	>5 to <15	≥15
Henagliflozin	≤5	>5 to <10	≥10
Ipragliflozin	≤100	>100 to <300	≥300
Licogliflozin	≤5	>5 to <10	≥10
Sotagliflozin	≤200	>200 to <400	≥400
Tofogliflozin	≤10	>10 to <40	≥40

Electronic Supplementary Table S3. Key characteristics of the included studies.

Study id	Indications	Interventions	Mean/Median age (years)	Male: Female (%)	HbA1c (%)	eGFR (ml/min/1.73m^2)	BMI (kg/m^2)	Duration of diabetes mellitus (years)	Dosing regimen
Anker 2021a [1]	HF without T2D	E	67.6	75.7: 24.3	5.8	62.7	27.2	No details	10 mg/day
		Placebo	66.3	74.6: 25.4	5.7	63	27		Not applicable
	HF/T2D	E	66.8	77.3: 22.7	7.4	61	28.8		10 mg/day
		Placebo	66.6	76.5: 23.5	7.4	61.4	28.6		Not applicable
Antlanger 2022 [2]	T2D/CKD stage III/IV	E	71	91.6: 8.4	6.9	33	31		10 mg/day
		Placebo	69	58.3: 41.7	6.8	37	28		Not applicable
	CKD stage III/IV	E	53	72.7: 27.3	5.4	37	26.3		10 mg/day
		Placebo	63	54.5: 45.5	5.6	29	27.7		Not applicable
Araki 2019 [3]	T1D	D/I	47.7	56.6: 43.4	8.4		25.3	14.6	5 mg/day
		D/I	48.9	44: 56	8.4		24.7	15.9	10 mg/day
Baker 2019 [4]	T1D	S	42.4	37.1: 62.9	8	90.6	27.4	22.2	75 mg/day
		S	47	57.1: 42.9	8.07	91.1	28	23.4	200 mg/day
		S	44.8	57.1: 42.9	8.05	88.7	29.4	24	400 mg/day
		Placebo	48.1	41.7: 58.3	7.95	87.4	31.8	26.9	Not applicable
Bhatt 2021a [5]	T2D/HF	S	69	67.4: 32.6	7.1	49.2	30.4		200 mg/day titrated to 400 mg/day
		Placebo	70	65.1: 34.9	7.2	50.5	31.1		Not applicable
Bhatt 2021b [6]	T2D/CKD	S	69	55.7: 44.3	8.3	44.4	31.9		200 mg/day titrated to 400 mg/day
		Placebo	69	54.5: 45.5	8.3	44.7	31.7		Not applicable
Bode 2021 [7]	T1D	S	22.8	48.8: 51.2	9.9	113.2	29.4	11.9	400 mg/day
		Placebo	21.7	45.2: 54.8	9.7	114.5	26.7	11.9	Not applicable
Brown 2020 [8]	T2D	D	64.25	62.5: 37.5	7.8	107.53	32.3	10	10 mg/day
		Placebo	66.74	52.9: 47.1	7.7	96.56	32.59	8.5	Not applicable

Buse 2018 [9]	T1D	S	46.6	47.9: 52.1	7.61	No details	29.81	25	200 mg/day
		S	46.4	45.8: 54.2	7.56		29.63	24	400 mg/day
		Placebo	45.2	51.1: 48.9	7.54		29.55	24.2	Not applicable
Cannon 2020 [10]	T2D	Er	64.4	70.3: 29.7	8.2	76.1	31.9	12.9	5 mg/day to one group and 15 mg/day to another
		Placebo	64.4	69.3: 30.7	8.2	75.7	32	13.1	Not applicable
Carbone 2020 [11]	T2D/HF	C	58	76.5: 23.5	8.3	74.7	34.5	No details	100 mg/day
		Sitagliptin	54.3	78.9: 21.1	8.3	83.3	38.8		100 mg/day
Cefalu 2013 [12] & Leiter 2015 [13]	T2D	C	56.4	52: 48	7.8	No details	31	6.5	100 mg/day
		C	55.8	50: 50	7.8		31.2	6.7	300 mg/day
Charaya 2022 [14]	HF	D	72.6	58: 42	No details	55.65	No details		10 mg/day
		Standard of care	74.2	52: 48		52.7			Not applicable
Cherney 2017 [15]	T2D/CKD	E	51.1	56.6: 43.4	8.02	106.4	28.3		25 mg/day
		Placebo	51.5	47.8: 52.2	8.08	106.1	27.9		Not applicable
		E	58.6	58.1: 41.9	7.94	76.2	29.4		25 mg/day
		Placebo	58.6	54.7: 45.3	8.03	76.2	29.2		Not applicable
		E	64.3	56.4: 43.6	7.99	46.8	30.3		25 mg/day
		Placebo	65.1	56.9: 43.1	7.98	46.8	30.5		Not applicable
		E	64.7	54.8: 45.2	7.94	24	31.4		25 mg/day
		Placebo	63.3	50: 50	8.14	22.7	28.7		Not applicable
Cherney 2020 [16]	CKD	D & Placebo (cross-over trial)	51	68: 32	5.6	58.3	28		D-10 mg/day
Cherney 2021 [17]	T2D/ CKD stage IV	S	66.8	47.8: 52.2	8.3	23.8	30.9	19.6	200 mg/day
		S	67.3	53.3: 46.7	8.3	23.9	32.1	18.5	400 mg/day
		Placebo	68	45.2: 54.8	8.4	24.1	31.7	20.7	Not applicable
Cherney 2023 [18]	T2D/ CKD stage III	S	69.6	54.4: 45.6	8.3	45.2	32.3	17.2	200 mg/day
		S	69.5	57.6: 42.4	8.3	45.1	32.4	16.3	400 mg/day
		Placebo	69.3	57.3: 42.7	8.3	44.8	32.5	17.7	Not applicable

Curovic 2022 [19]	T2D	D & Placebo (cross-over trial)	63	88: 12	8.8	89	33.7	15.4	D-10 mg/day
Damman 2020 [20]	HF	E	79	60: 40	No details	55	No details		10 mg/day
		Placebo	73	74: 26		55			Not applicable
Danne 2018 [21]	T1D	S	42.3	53.3: 46.7	7.74		27.97	18.2	200 mg/day
		S	41.7	51.6: 49.4	7.71		27.85	18.9	400 mg/day
		Placebo	39.7	51.9: 48.1	7.79		27.5	18.1	Not applicable
de Boer 2020 [22]	T2D/HF	L	70	93.3: 6.7		76.3	33.3		2.5 mg/day
		L	72.5	75: 25		61.3	31.9		10 mg/day
		L	66	80: 20		69.8	32		50 mg/day
		E	68.5	67.7: 33.3		63.5	31.2		25 mg/day
		Placebo	71	57.6: 42.4		66.5	31.3		Not applicable
Fioretto 2018 [23]	T2D/ CKD stage III	D	65.3	56.9: 43.1	8.33	53.3	32.6	14.3	10 mg/day
		Placebo	66.2	56.5: 43.5	8.03	53.6	31.6	14.5	Not applicable
Garcia-Tirado 2022 [24]	T1D	E/Insulin	40	27.8: 72.2	6.7	91.5	30	21	E-5 mg/day
		Insulin	42	35.3: 64.7	7.1	86.5	29	21	According to the capillary blood glucose
Greeviroj 2023 [25]	Non-diabetic obese with albuminuria	C	44.56	43.75: 56.25	No details	97.05	36.39	Not applicable	100 mg/day
		Placebo	50: 50	50: 50		104.45	36.63		Not applicable
Griffin 2020 [26]	T2D/HF	E & Placebo	60	75: 25	7.1	69.1	37	No details	E-10 mg/day
Haidar 2022 [27]	T1D	E & Placebo	38	44: 56	7.7		29.5	24	E-25 mg/day

Halvorsen 2023 [28]	T2D	B	56	63.3: 36.7	8.66		29.7	9.31	20 mg/day
		Placebo	55.6	59.1: 40.9	8.57		30	8.88	Not applicable
Heerspink 2020 [29]	CKD	D	61.8	67.1: 32.9	No details	43.2	29.4	No details	10 mg/day
		Placebo	61.9	66.7: 33.3		43	29.6		Not applicable
Henry 2015 [30]	T1D	C	42	59: 41	7.9	97.4	28	22	100 mg/day
		C	42.8	55.6: 44.4	8	95.8	28.1	21.9	300 mg/day
		Placebo	42	53.8: 46.2	7.9	96	28	23.3	Not applicable
Henry 2015b [31]	T1D	D	33.7	38.5: 61.5	8.21	No details	25.1	20.1	1 mg/day
		D	35.7	73.3: 26.7	8.45		24.8	21.7	2.5 mg/day
		D	34.8	57.1: 42.9	8.5		23.4	17.2	5 mg/day
		D	37.5	53.3: 46.7	8.39		25.8	18.1	10 mg/day
		Placebo	34.5	61.5: 38.5	8.75		25.3	16.2	Not applicable
Hollander 2017 [32]	Overweight/ Obese without T2D	C	45.2	19: 81	5.6	95.4	37.3	No details	300 mg/day
		Phentermine	46.4	18.8: 81.2	5.6	95.3	37		15 mg/day
		C/Phentermine	46.3	16.9: 83.1	5.6	97.4	36.8		C-300 mg/day and phentermine-15 mg/day
		Placebo	44.8	18.3: 81.7	5.6	95.2	38		Not applicable
Ikeda 2015 [33]	T2D	T	53.3	51.5: 48.5	7.99		31.33	4.88	2.5 mg/day
		T	54.8	47.7: 52.3	8.01		30.56	5.01	5 mg/day
		T	54.5	51.5: 48.5	8		30.4	5.77	10 mg/day
		T	56.3	67.2: 32.8	7.92		30.09	5.21	20 mg/day
		T	57.5	46.3: 53.7	7.92		30.36	6.44	40 mg/day
		Placebo	53.9	54.5: 45.5	7.88		30.37	5.98	Not applicable
Ito 2017 [34]	T2D/	I	57.3	44: 56	8.5	No details	30.7	8.7	50 mg/day

	NAFLD	Pioglitazone	59.1	53: 47	8.3		29.9	9.5	15-30 mg/day
Ito 2021 [35]	T2D	D	55.9	72.7: 27.3	7.86		27.7	No details	5 mg/day
		Sitagliptin	53.8	60: 40	8.01		27.1		50 mg/day
		Metformin	57.5	90: 10	7.93		26.7		1 g/day
Ji 2023 [36]	T2D	E	59.9	58.9: 41.1	8.64	83.02	26.41	14.74	10 mg/day
		E	60.7	54.8: 45.2	8.64	81.04	25.4	15.05	25 mg/day
		Placebo	60.1	49.3: 50.7	8.64	84.51	25.87	14.14	Not applicable
Jurgens 2021 [37]	T2D	E	66	76: 24	7.7	79.5	31.9	14	25 mg/day
		Placebo	67	84: 16	7.5	79.3	29.8	15	Not applicable
Kadowaki 2017 [38]	T2D	C/Teneligliptin	58.4	77.1: 22.9	8.18	84.7	25.53	8.34	C-100 mg/day & teneligiptin-20 mg/day
		Teneligliptin	56	77.9: 22.1	7.87	83.9	26.44	6.5	20 mg/day
Kashyap 2020 [39]	T2D with bariatric surgery	C	58	36.4: 63.6	7.2	No details	39.6	No details	100 to 300 mg/day
		Placebo	44	20: 80	8.2		37.9		Not applicable
Katsiadas 2022 [40]	T2D	D	68.11	83.9: 16.1	7.45	72	27.6		10 mg/day
		Control	71.87	71.87: 28.13	7.2	68.8	27.9		Not specified
Kawamori 2018 [41]	T2D	E	60	78: 22	8.27	89.3	26	9	10 mg/day until 24 weeks and titrated to 25 mg/day based on HbA1c until 52 weeks
		Placebo	59.8	77.4: 22.6	8.36	86.3	26.6	8.7	Not applicable
Kuchay 2023 [42]	T2D	D	59	89.6: 10.4	7.8	No details	25.3	No details	10 mg/day
		Placebo	63	85.6: 14.4	7.6		25.7		Not applicable
Laffel 2023 [43]	T2D	E	14.4	37: 63	8	130.09	35.54		10-25 mg/day
		Linagliptin	14.6	42: 58	8.05	135.11	36.5		5 mg/day
		Placebo	14.6	36: 64	8.05	124.28	36.07		Not applicable
Lewinski 2022 [44]	AMI	E	57	82: 18	5.6	92	27.7		10 mg/day
		Placebo	57	82: 18	5.7	91	27.2		Not applicable
	T1D	D	42.7	43.5: 56.5	8.45	No details	27.27	19.35	5 mg/day
		D	42.4	44.8: 55.2	8.43		27.8	19.45	10 mg/day

Mathieu 2018 [45] & Mathieu 2020 [46]		Placebo	43	43.8: 56.2	8.43		27.6	18.98	Not applicable
McMurray 2019 [47]	HF	D	66.2	76.2: 23.8	No details	66	28.2	No details	10 mg/day
		Placebo	66.5	77: 23		65.5	28.1		Not applicable
Nishimura 2015 [48]	T2D	E	64.8	70: 30	7.9	76.5	24.1	No details	10 mg/day
		E	62.6	84.2: 15.8	7.73	80.7	24		25 mg/day
		Placebo	60.7	81: 19	8	82.6	24.9		Not applicable
Op den Kamp 2021 [49]	T2D	D & Placebo	64.2	79.2: 20.8	6.9	141	28.1	8	Not applicable
Perkovic 2019 [50]	T2D	C	62.9	65.4: 34.6	8.3	56.3	31.4	15.5	100 mg/day
		Placebo	63.2	66.7: 33.3	8.3	56	31.3	16	Not applicable
Pollock 2019 [51]	T2D	D	64.7	70: 30	8.44	50.2	30.19	17.55	10 mg/day
		D/Saxagliptin	64	71: 29	8.2	49	30.81	18.43	D-10 mg/day & saxagliptin-2.5 mg/day
		Placebo	64.7	71: 29	8.57	47.7	30.34	17.71	Not applicable
Rau 2021 [52]	T2D	E	62.8	80: 20	No details	77	31.4		10 mg/day
		Placebo	61.2	81.8: 18.2		88	31.2		Not applicable
Reis 2022 [53]	HF	D	60.3	85: 15	No details	68.7	No details		10 mg/day
		Control	61.7	80: 20		72.5			Not applicable
Ridderstrale 2014 [54] & Ridderstrale 2018 [55]	T2D	E	56.2	56: 44	7.92	No details	30	No details	25 mg/day
		Glimepiride	55.7	54: 46	7.92		30.3		1-4 mg/day
Sands 2015 [56]	T1D	S	45.5	50: 50	7.94	No details	27.1	16.8	400 mg/day
		Placebo	34	47: 53	7.98		26.2	18.5	Not applicable
Sargeant 2022 [57]	T2D	E	63	53: 47	6.85	90	31.3	6.25	25 mg/day
		Placebo	63	76.5: 23.5	6.95	90	32.5	5.5	Not applicable

Satirapoj 2019 [58]	T2D	D	55.9	60.7: 39.3	8.7	No details	28.4	8.5	10 mg/day
		Control	59.9	27: 73	8.6		27.9	9	Not applicable
Shimada 2018 [59]	T2D	E	44.2	38.5: 61.5	8.02	88	24.4	16.8	2.5 mg/day
		E	44.5	33.3: 66.7	8.12	87	22.68	14.3	10 mg/day
		E	46.6	66.7: 33.3	7.89	88.8	22.6	20.8	25 mg/day
		Placebo	43.9	45.5: 54.5	8.23	95.1	23.7	14.8	Not applicable
Softeland 2017 [60]	T2D	E	54.3	60.6: 39.4	7.97	90.8	31.2	No details	10 mg/day
		E	55.4	64.5: 35.5	7.97	93.4	29.9		25 mg/day
		Placebo	55.9	55.6: 44.4	7.97	92.7	29.6		Not applicable
Solomon 2022 [61]	HF	D	71.8	56.4: 43.6	No details	61	29.8		10 mg/day
		Placebo	71.5	55.8: 44.2		61	29.9		Not applicable
Terauchi 2019 [62]	T2D	E	55.6	81.3: 18.7	8.83	89.3	28		10 mg/day
		E	58.9	66.7: 33.3	8.68	82	27.7		25 mg/day
Van Meijel 2020 [63]	T1D	D & Placebo (crossover trial)	49.7	40: 60	7.5	No details	25.1	24.1	D- 10 mg/day
Veelen 2023 [64]	Pre-diabetic	D & Placebo (crossover trial)	66.3	57.1: 42.9	5.5	99	30	No details	D- 10 mg/day
Vilsboll 2019 [65] & Vilsboll 2020 [66]	T2D	D/Saxagliptin/Metformin	55.7	54.3: 45.7	9	94.6	32.5	9.6	D-10 mg/day, saxagliptin-5 mg/day & metformin ≥ 1.5 g/day
		Insulin/Metformin	55.3	53.6: 46.4	9.1	97.3	32	9.3	Insulin-according to blood sugar & metformin ≥ 1.5 g/day
Watada 2018 [67]	T1D	D	37	57.1: 42.9	7.9	91.6	23	15.9	5 mg/day
		D	37.1	50: 50	7.9	94.6	22.2	14.7	10 mg/day
		Placebo	42.6	21.4: 78.6	8.1	95.4	22.9	16.9	Not applicable
Weng 2021 [68]	T2D	H	54.3	63.6: 36.4	8.5	125.4	25.5	5.5	5 mg/day
		H	54.7	63.1: 36.9	8.4	128.9	25.6	6.4	10 mg/day
		Placebo	55.3	57.8: 42.2	8.5	128	25.4	6.6	Not applicable

Wiviott 2019 [69] & Cahn 2020 [70]	T2D	D	63.9	63.1: 36.9	8.3	85.4	32.1	11	10 mg/day
		Placebo	64	62.1: 37.9	8.3	85.1	32	10	Not applicable
EMPA-KIDNEY 2023 [71]	CKD	E	63.9	56.8: 33.2	No details	37.4	29.7	No details	10 mg/day
		Placebo	63.8	56.9: 33.1		37.3	29.8		Not applicable
Rosenstock 2018 [72] & Gallo 2019 [73]	T2D	Er	56.6	46.9: 53.1	8.1	88.9	30.8	7.9	5 mg/day
		Er	56.9	45.4: 54.6	8.1	91	31.1	8.1	15 mg/day
		Placebo	56.5	46.9: 53.1	8.2	91.6	30.7	8	Not applicable

T2D-Type 2 diabetes; T1D-Type 1 diabetes; HF-Heart failure; AMI-Acute myocardial infarction; CKD-chronic kidney disease; and NAFLD-Non-alcoholic fatty liver disease.

B-Bexagliflozin; E-Empagliflozin; D-Dapagliflozin; C-Canagliflozin; Er-Ertugliflozin; H-Henagliflozin; I-Ipragliflozin; L-Licogliflozin; S-Sotagliflozin; and T-Tofogliflozin.

D/I-Dapagliflozin/Insulin; Placebo/I-Placebo/Insulin; OD-Once daily; and BD-twice daily.

References of included studies:

1. Anker SD, Butler J, Filippatos G, Khan MS, Marx N, Lam CSP, Schnaidt S, Ofstad AP, Brueckmann M, Jamal W, Bocchi EA, Ponikowski P, Perrone SV, Januzzi JL, Verma S, Böhm M, Ferreira JP, Pocock SJ, Zannad F, Packer M. Effect of Empagliflozin on Cardiovascular and Renal Outcomes in Patients With Heart Failure by Baseline Diabetes Status: Results From the EMPEROR-Reduced Trial. Circulation. 2021 Jan 26;143(4):337-349.

2. Antlanger M, Domenig O, Kaltenecker CC, Kovarik JJ, Rathkolb V, Müller MM, Schwaiger E, Hecking M, Poglitsch M, Säemann MD, Kopecky C. Combined sodium glucose co-transporter-2 inhibitor and angiotensin-converting enzyme inhibition upregulates the renin-angiotensin system in chronic kidney disease with type 2 diabetes: Results of a randomized, double-blind, placebo-controlled exploratory trial. Diabetes Obes Metab. 2022 May;24(5):816-826.

3. Araki E, Watada H, Uchigata Y, Tomonaga O, Fujii H, Ohashi H, Okabe T, Asano M, Thoren F, Kim H, Yajima T, Langkilde AM. Efficacy and safety of dapagliflozin in Japanese patients with inadequately controlled type 1 diabetes (DEPICT-5): 52-week results from a randomized, open-label, phase III clinical trial. Diabetes Obes Metab. 2020 Apr;22(4):540-548.

4. Baker C, Wason S, Banks P, Sawhney S, Chang A, Danne T, Gesty-Palmer D, Kushner JA, McGuire DK, Mikell F, O'Neill M, Peters AL, Strumph P. Dose-dependent glycometabolic effects of sotagliflozin on type 1 diabetes over 12 weeks: The inTandem4 trial. Diabetes Obes Metab. 2019 Nov;21(11):2440-2449.

5. Bhatt DL, Szarek M, Steg PG, Cannon CP, Leiter LA, McGuire DK, Lewis JB, Riddle MC, Voors AA, Metra M, Lund LH, Komajda M, Testani JM, Wilcox CS, Ponikowski P, Lopes RD, Verma S, Lapuerta P, Pitt B; SOLOIST-WHF Trial Investigators. Sotagliflozin in Patients with Diabetes and Recent Worsening Heart Failure. N Engl J Med. 2021 Jan 14;384(2):117-128.

6. Bhatt DL, Szarek M, Pitt B, Cannon CP, Leiter LA, McGuire DK, Lewis JB, Riddle MC, Inzucchi SE, Kosiborod MN, Cherney DZI, Dwyer JP, Scirica BM, Bailey CJ, Díaz R, Ray KK, Udell JA, Lopes RD, Lapuerta P, Steg PG; SCORED Investigators. Sotagliflozin in Patients with Diabetes and Chronic Kidney Disease. N Engl J Med. 2021 Jan 14;384(2):129-139.

7. Bode BW, Cengiz E, Wadwa RP, Banks P, Danne T, Kushner JA, McGuire DK, Peters AL, Strumph P, Sawhney S. Effects of Sotagliflozin Combined with Intensive Insulin Therapy in Young Adults with Poorly Controlled Type 1 Diabetes: The JDRF Sotagliflozin Study. Diabetes Technol Ther. 2021 Jan;23(1):59-69.

8. Brown AJM, Gandy S, McCrimmon R, Houston JG, Struthers AD, Lang CC. A randomized controlled trial of dapagliflozin on left ventricular hypertrophy in people with type two diabetes: the DAPA-LVH trial. Eur Heart J. 2020 Sep 21;41(36):3421-3432.

9. Buse JB, Garg SK, Rosenstock J, Bailey TS, Banks P, Bode BW, Danne T, Kushner JA, Lane WS, Lapuerta P, McGuire DK, Peters AL, Reed J, Sawhney S, Strumph P. Sotagliflozin in Combination With Optimized Insulin Therapy in Adults With Type 1 Diabetes: The North American inTandem1 Study. Diabetes Care. 2018 Sep;41(9):1970-1980.

10. Cannon CP, Pratley R, Dagogo-Jack S, Mancuso J, Huyck S, Masiukiewicz U, Charbonnel B, Frederich R, Gallo S, Cosentino F, Shih WJ, Gantz I, Terra SG, Cherney DZI, McGuire DK; VERTIS CV Investigators. Cardiovascular Outcomes with Ertugliflozin in Type 2 Diabetes. N Engl J Med. 2020 Oct 8;383(15):1425-1435.

11. Carbone S, Billingsley HE, Canada JM, Bressi E, Rotelli B, Kadariya D, Dixon DL, Markley R, Trankle CR, Cooke R, Rao K, B Shah K, Medina de Chazal H, Chiabrando JG, Vecchié A, Dell M, L Mihalick V, Bogaev R, Hart L, Van Tassell BW, Arena R, Celi FS, Abbate A. The effects of canagliflozin compared to sitagliptin on cardiorespiratory fitness in type 2 diabetes mellitus and heart failure with reduced ejection fraction: The CANA-HF study. Diabetes Metab Res Rev. 2020 Nov;36(8):e3335.

12. Cefalu WT, Leiter LA, Yoon KH, Arias P, Niskanen L, Xie J, Balis DA, Canovatchel W, Meininger G. Efficacy and safety of canagliflozin versus glimepiride in patients with type 2 diabetes inadequately controlled with metformin (CANTATA-SU): 52 week results from a randomised, double-blind, phase 3 non-inferiority trial. Lancet. 2013 Sep 14;382(9896):941-50.

13. Leiter LA, Yoon KH, Arias P, Langslet G, Xie J, Balis DA, Millington D, Vercruysse F, Canovatchel W, Meininger G. Canagliflozin provides durable glycemic improvements and body weight reduction over 104 weeks versus glimepiride in patients with type 2 diabetes on metformin: a randomized, double-blind, phase 3 study. Diabetes Care. 2015 Mar;38(3):355-64.

14. Cefalu WT, Leiter LA, de Bruin TW, Gause-Nilsson I, Sugg J, Parikh SJ. Dapagliflozin's Effects on Glycemia and Cardiovascular Risk Factors in High-Risk Patients With Type 2 Diabetes: A 24-Week, Multicenter, Randomized, Double-Blind, Placebo-Controlled Study With a 28-Week Extension. Diabetes Care. 2015 Jul;38(7):1218-27.

15. Cherney DZI, Cooper ME, Tikkanen I, Pfarr E, Johansen OE, Woerle HJ, Broedl UC, Lund SS. Pooled analysis of Phase III trials indicate contrasting influences of renal function on blood pressure, body weight, and HbA1c reductions with empagliflozin. Kidney Int. 2018 Jan;93(1):231-244.

16. Cherney DZI, Dekkers CCJ, Barbour SJ, Cattran D, Abdul Gafor AH, Greasley PJ, Laverman GD, Lim SK, Di Tanna GL, Reich HN, Vervloet MG, Wong MG, Gansevoort RT, Heerspink HJL; DIAMOND investigators. Effects of the SGLT2 inhibitor dapagliflozin on proteinuria in non-diabetic patients with chronic kidney disease (DIAMOND): a randomised, double-blind, crossover trial. Lancet Diabetes Endocrinol. 2020 Jul;8(7):582-593.

17. Cherney DZI, Ferrannini E, Umpierrez GE, Peters AL, Rosenstock J, Carroll AK, Lapuerta P, Banks P, Agarwal R. Efficacy and safety of sotagliflozin in patients with type 2 diabetes and severe renal impairment. Diabetes Obes Metab. 2021 Dec;23(12):2632-2642.

18. Cherney DZI, Ferrannini E, Umpierrez GE, Peters AL, Rosenstock J, Powell DR, Davies MJ, Banks P, Agarwal R. Efficacy and safety of sotagliflozin in patients with type 2 diabetes and stage 3 chronic kidney disease. Diabetes Obes Metab. 2023 Jun;25(6):1646-1657.

19. Curovic VR, Eickhoff MK, Rönkkö T, Frimodt-Møller M, Hansen TW, Mischak H, Rossing P, Ahluwalia TS, Persson F. Dapagliflozin Improves the Urinary Proteomic Kidney-Risk Classifier CKD273 in Type 2 Diabetes with Albuminuria: A Randomized Clinical Trial. Diabetes Care. 2022 Nov 1;45(11):2662-2668.

20. Damman K, Beusekamp JC, Boorsma EM, Swart HP, Smilde TDJ, Elvan A, van Eck JWM, Heerspink HJL, Voors AA. Randomized, double-blind, placebo-controlled, multicentre pilot study on the effects of empagliflozin on clinical outcomes in patients with acute decompensated heart failure (EMPA-RESPONSE-AHF). Eur J Heart Fail. 2020 Apr;22(4):713-722.

21. Danne T, Cariou B, Banks P, Brandle M, Brath H, Franek E, Kushner JA, Lapuerta P, McGuire DK, Peters AL, Sawhney S, Strumph P. HbA1c and Hypoglycemia Reductions at 24 and 52 Weeks With Sotagliflozin in Combination With Insulin in Adults With Type 1 Diabetes: The European inTandem2 Study. Diabetes Care. 2018 Sep;41(9):1981-1990.

22. de Boer RA, Núñez J, Kozlovski P, Wang Y, Proot P, Keefe D. Effects of the dual sodium-glucose linked transporter inhibitor, licogliflozin vs placebo or empagliflozin in patients with type 2 diabetes and heart failure. Br J Clin Pharmacol. 2020 Jul;86(7):1346-1356.

23. Fioretto P, Del Prato S, Buse JB, Goldenberg R, Giorgino F, Reyner D, Langkilde AM, Sjöström CD, Sartipy P; DERIVE Study Investigators. Efficacy and safety of dapagliflozin in patients with type 2 diabetes and moderate renal impairment (chronic kidney disease stage 3A): The DERIVE Study. Diabetes Obes Metab. 2018 Nov;20(11):2532-2540.

24. Garcia-Tirado J, Farhy L, Nass R, Kollar L, Clancy-Oliveri M, Basu R, Kovatchev B, Basu A. Automated Insulin Delivery with SGLT2i Combination Therapy in Type 1 Diabetes. Diabetes Technol Ther. 2022 Jul;24(7):461-470.

25. Greeviroj P, Puapatanakul P, Phannajit J, Takkavatakarn K, Kittanamongkolchai W, Boonchaya-Anant P, Katavetin P, Praditpornsilpa K, Eiam-Ong S, Susantitaphong P. Effect of canagliflozin in non-diabetic obese patients with albuminuria: A randomized, double-blind, placebo-controlled trial. Clin Nephrol. 2023 Nov;100(5):224-230.

26. Griffin M, Rao VS, Ivey-Miranda J, Fleming J, Mahoney D, Maulion C, Suda N, Siwakoti K, Ahmad T, Jacoby D, Riello R, Bellumkonda L, Cox Z, Collins S, Jeon S, Turner JM, Wilson FP, Butler J, Inzucchi SE, Testani JM. Empagliflozin in Heart Failure: Diuretic and Cardiorenal Effects. Circulation. 2020 Sep 15;142(11):1028-1039.

27. Haidar A, Lovblom LE, Cardinez N, Gouchie-Provencher N, Orszag A, Tsoukas MA, Falappa CM, Jafar A, Ghanbari M, Eldelekli D, Rutkowski J, Yale JF, Perkins BA. Empagliflozin add-on therapy to closed-loop insulin delivery in type 1 diabetes: a 2 × 2 factorial randomized crossover trial. Nat Med. 2022 Jun;28(6):1269-1276.

28. Halvorsen YD, Conery AL, Lock JP, Zhou W, Freeman MW. Bexagliflozin as an adjunct to metformin for the treatment of type 2 diabetes in adults: A 24-week, randomized, double-blind, placebo-controlled trial. Diabetes Obes Metab. 2023 Oct;25(10):2954-2962.

29. Heerspink HJL, Stefánsson BV, Correa-Rotter R, Chertow GM, Greene T, Hou FF, Mann JFE, McMurray JJV, Lindberg M, Rossing P, Sjöström CD, Toto RD, Langkilde AM, Wheeler DC; DAPA-CKD Trial Committees and Investigators. Dapagliflozin in Patients with Chronic Kidney Disease. N Engl J Med. 2020 Oct 8;383(15):1436-1446.

30. Henry RR, Thakkar P, Tong C, Polidori D, Alba M. Efficacy and Safety of Canagliflozin, a Sodium-Glucose Cotransporter 2 Inhibitor, as Add-on to Insulin in Patients With Type 1 Diabetes. Diabetes Care. 2015 Dec;38(12):2258-65.

31. Henry RR, Rosenstock J, Edelman S, Mudaliar S, Chalamandaris AG, Kasichayanula S, Bogle A, Iqbal N, List J, Griffen SC. Exploring the potential of the SGLT2 inhibitor dapagliflozin in type 1 diabetes: a randomized, double-blind, placebo-controlled pilot study. Diabetes Care. 2015 Mar;38(3):412-9.

32. Hollander P, Bays HE, Rosenstock J, Frustaci ME, Fung A, Vercruysse F, Erondu N. Coadministration of Canagliflozin and Phentermine for Weight Management in Overweight and Obese Individuals Without Diabetes: A Randomized Clinical Trial. Diabetes Care. 2017 May;40(5):632-639.

33. Ikeda S, Takano Y, Cynshi O, Tanaka R, Christ AD, Boerlin V, Beyer U, Beck A, Ciorciaro C, Meyer M, Kadowaki T. A novel and selective sodium-glucose cotransporter-2 inhibitor,

tofogliflozin, improves glycaemic control and lowers body weight in patients with type 2 diabetes mellitus. Diabetes Obes Metab. 2015 Oct;17(10):984-93.

34. Ito D, Shimizu S, Inoue K, Saito D, Yanagisawa M, Inukai K, Akiyama Y, Morimoto Y, Noda M, Shimada A. Comparison of Ipragliflozin and Pioglitazone Effects on Nonalcoholic Fatty Liver Disease in Patients With Type 2 Diabetes: A Randomized, 24-Week, Open-Label, Active-Controlled Trial. Diabetes Care. 2017 Oct;40(10):1364-1372.

35. Ito D, Inoue K, Saito D, Hamaguchi K, Kaneko K, Sumita T, Inukai K, Inoue I, Shimada A. Effects of Dapagliflozin Compared with Sitagliptin and Metformin in Drug-Naïve Japanese Patients with Type 2 Diabetes: A 12-Week, Open-Label, Randomized, Active-Controlled Trial. Diabetes Ther. 2021 Dec;12(12):3201-3215.

36. Ji L, Lu Y, Li Q, Fu L, Luo Y, Lei T, Li L, Ye S, Shi B, Li X, Meinicke T. Efficacy and safety of empagliflozin in combination with insulin in Chinese patients with type 2 diabetes and insufficient glycaemic control: A phase III, randomized, double-blind, placebo-controlled, parallel study. Diabetes Obes Metab. 2023 Jul;25(7):1839-1848.

37. Jürgens M, Schou M, Hasbak P, Kjær A, Wolsk E, Zerahn B, Wiberg M, Brandt-Jacobsen NH, Gæde P, Rossing P, Faber J, Inzucchi SE, Gustafsson F, Kistorp C. Effects of Empagliflozin on Myocardial Flow Reserve in Patients With Type 2 Diabetes Mellitus: The SIMPLE Trial. J Am Heart Assoc. 2021; 10(15): e020418.

38. Kadowaki T, Inagaki N, Kondo K, Nishimura K, Kaneko G, Maruyama N, Nakanishi N, Iijima H, Watanabe Y, Gouda M. Efficacy and safety of canagliflozin as add-on therapy to teneligliptin in Japanese patients with type 2 diabetes mellitus: Results of a 24-week, randomized, double-blind, placebo-controlled trial. Diabetes Obes Metab. 2017 Jun;19(6):874-882.

39. Kashyap SR, Kheniser K, Aminian A, Schauer P, Le Roux C, Burguera B. Double-blinded, randomized, and controlled study on the effects of canagliflozin after bariatric surgery: A pilot study. Obes Sci Pract. 2020 Mar 17;6(3):255-263.

40. Katsiadas N, Xanthopoulos A, Giamouzis G, Skoularigkis S, Skopeliti N, Moustaferi E, Ioannidis I, Patsilinakos S, Triposkiadis F, Skoularigis J. The effect of SGLT-2i administration on red blood cell distribution width in patients with heart failure and type 2 diabetes mellitus: A randomized study. Front Cardiovasc Med. 2022 Sep 29;9:984092.

41. Kawamori R, Haneda M, Suzaki K, Cheng G, Shiki K, Miyamoto Y, Solimando F, Lee C, Lee J, George J. Empagliflozin as add-on to linagliptin in a fixed-dose combination in Japanese patients with type 2 diabetes: Glycaemic efficacy and safety profile in a 52-week, randomized, placebo-controlled trial. Diabetes Obes Metab. 2018 Sep;20(9):2200-2209.

42. Kuchay MS, Khatana P, Mishra M, Surendran P, Kaur P, Wasir JS, Gill HK, Singh A, Jain R, Kohli C, Bakshi G, Radhika V, Saheer S, Singh MK, Mishra SK. Dapagliflozin for inpatient hyperglycemia in cardiac surgery patients with type 2 diabetes: randomised controlled trial (Dapa-Hospital trial). Acta Diabetol. 2023 Nov;60(11):1481-1490.

43. Laffel LM, Danne T, Klingensmith GJ, Tamborlane WV, Willi S, Zeitler P, Neubacher D, Marquard J; DINAMO Study Group. Efficacy and safety of the SGLT2 inhibitor empagliflozin versus placebo and the DPP-4 inhibitor linagliptin versus placebo in young people with type 2 diabetes (DINAMO): a multicentre, randomised, double-blind, parallel group, phase 3 trial. Lancet Diabetes Endocrinol. 2023 Mar;11(3):169-181.

44. von Lewinski D, Kolesnik E, Tripolt NJ, Pferschy PN, Benedikt M, Wallner M, Alber H, Berger R, Lichtenauer M, Saely CH, Moertl D, Auersperg P, Reiter C, Rieder T, Siller-Matula JM, Gager GM, Hasun M, Weidinger F, Pieber TR, Zechner PM, Herrmann M, Zirlik A, Holman RR, Oulhaj A, Sourij H. Empagliflozin in acute myocardial infarction: the EMMY trial. Eur Heart J. 2022 Nov 1;43(41):4421-4432.

45. Mathieu C, Dandona P, Gillard P, Senior P, Hasslacher C, Araki E, Lind M, Bain SC, Jabbour S, Arya N, Hansen L, Thorén F, Langkilde AM; DEPICT-2 Investigators. Efficacy and Safety of Dapagliflozin in Patients With Inadequately Controlled Type 1 Diabetes (the DEPICT-2 Study): 24-Week Results From a Randomized Controlled Trial. Diabetes Care. 2018 Sep;41(9):1938-1946.

46. Mathieu C, Rudofsky G, Phillip M, Araki E, Lind M, Arya N, Thorén F, Scheerer MF, Iqbal N, Dandona P. Long-term efficacy and safety of dapagliflozin in patients with inadequately controlled type 1 diabetes (the DEPICT-2 study): 52-week results from a randomized controlled trial. Diabetes Obes Metab. 2020 Sep;22(9):1516-1526.

47. McMurray JJV, Solomon SD, Inzucchi SE, Køber L, Kosiborod MN, Martinez FA, Ponikowski P, Sabatine MS, Anand IS, Bělohlávek J, Böhm M, Chiang CE, Chopra VK, de Boer RA, Desai AS, Diez M, Drozdz J, Dukát A, Ge J, Howlett JG, Katova T, Kitakaze M, Ljungman CEA, Merkely B, Nicolau JC, O'Meara E, Petrie MC, Vinh PN, Schou M, Tereshchenko S, Verma S, Held C, DeMets DL, Docherty KF, Jhund PS, Bengtsson O, Sjöstrand M, Langkilde AM; DAPA-HF Trial Committees and Investigators. Dapagliflozin in Patients with Heart Failure and Reduced Ejection Fraction. N Engl J Med. 2019 Nov 21;381(21):1995-2008.

48. Nishimura R, Tanaka Y, Koiwai K, Inoue K, Hach T, Salsali A, Lund SS, Broedl UC. Effect of empagliflozin monotherapy on postprandial glucose and 24-hour glucose variability in Japanese patients with type 2 diabetes mellitus: a randomized, double-blind, placebo-controlled, 4-week study. Cardiovasc Diabetol. 2015 Jan 30;14:11.

49. Op den Kamp YJM, de Ligt M, Dautzenberg B, Kornips E, Esterline R, Hesselink MKC, Hoeks J, Schrauwen-Hinderling VB, Havekes B, Oscarsson J, Phielix E, Schrauwen P. Effects of the SGLT2 Inhibitor Dapagliflozin on Energy Metabolism in Patients With Type 2 Diabetes: A Randomized, Double-Blind Crossover Trial. Diabetes Care. 2021 Jun;44(6):1334-1343.

50. Perkovic V, Jardine MJ, Neal B, Bompoint S, Heerspink HJL, Charytan DM, Edwards R, Agarwal R, Bakris G, Bull S, Cannon CP, Capuano G, Chu PL, de Zeeuw D, Greene T, Levin A, Pollock C, Wheeler DC, Yavin Y, Zhang H, Zinman B, Meininger G, Brenner BM, Mahaffey KW; CREDENCE Trial Investigators. Canagliflozin and Renal Outcomes in Type 2 Diabetes and Nephropathy. N Engl J Med. 2019 Jun 13;380(24):2295-2306.

51. Pollock C, Stefánsson B, Reyner D, Rossing P, Sjöström CD, Wheeler DC, Langkilde AM, Heerspink HJL. Albuminuria-lowering effect of dapagliflozin alone and in combination with saxagliptin and effect of dapagliflozin and saxagliptin on glycaemic control in patients with type 2 diabetes and chronic kidney disease (DELIGHT): a randomised, double-blind, placebo-controlled trial. Lancet Diabetes Endocrinol. 2019 Jun;7(6):429-441.

52. Rau M, Thiele K, Hartmann NK, Schuh A, Altiok E, Möllmann J, Keszei AP, Böhm M, Marx N, Lehrke M. Empagliflozin does not change cardiac index nor systemic vascular resistance but rapidly improves left ventricular filling pressure in patients with type 2 diabetes: a randomized controlled study. Cardiovasc Diabetol. 2021 Jan 7;20(1):6.

54. Ridderstråle M, Andersen KR, Zeller C, Kim G, Woerle HJ, Broedl UC; EMPA-REG H2H-SU trial investigators. Comparison of empagliflozin and glimepiride as add-on to metformin in patients with type 2 diabetes: a 104-week randomised, active-controlled, double-blind, phase 3 trial. Lancet Diabetes Endocrinol. 2014 Sep;2(9):691-700. doi: 10.1016/S2213-8587(14)70120-2.

55. Ridderstråle M, Rosenstock J, Andersen KR, Woerle HJ, Salsali A; EMPA-REG H2H-SU trial investigators. Empagliflozin compared with glimepiride in metformin-treated patients with type 2 diabetes: 208-week data from a masked randomized controlled trial. Diabetes Obes Metab. 2018 Dec;20(12):2768-2777.

56. Rosenstock J, Aggarwal N, Polidori D, Zhao Y, Arbit D, Usiskin K, Capuano G, Canovatchel W; Canagliflozin DIA 2001 Study Group. Dose-ranging effects of canagliflozin, a sodium-glucose cotransporter 2 inhibitor, as add-on to metformin in subjects with type 2 diabetes. Diabetes Care. 2012 Jun;35(6):1232-8.

57. Sargeant JA, King JA, Yates T, Redman EL, Bodicoat DH, Chatterjee S, Edwardson CL, Gray LJ, Poulin B, Waheed G, Waller HL, Webb DR, Willis SA, Wilding JPH, Khunti K, Stensel DJ, Davies MJ. The effects of empagliflozin, dietary energy restriction, or both on appetite-regulatory gut peptides in individuals with type 2 diabetes and overweight or obesity: The SEESAW

randomized, double-blind, placebo-controlled trial. Diabetes Obes Metab. 2022 Aug;24(8):1509-1521.

58. Satirapoj B, Korkiatpitak P, Supasyndh O. Effect of sodium-glucose cotransporter 2 inhibitor on proximal tubular function and injury in patients with type 2 diabetes: a randomized controlled trial. Clin Kidney J. 2019 Jan 4;12(3):326-332.

59. Shimada A, Hanafusa T, Yasui A, Lee G, Taneda Y, Sarashina A, Shiki K, George J, Soleymanlou N, Marquard J. Empagliflozin as adjunct to insulin in Japanese participants with type 1 diabetes: Results of a 4-week, double-blind, randomized, placebo-controlled phase 2 trial. Diabetes Obes Metab. 2018 Sep;20(9):2190-2199.

60. Søfteland E, Meier JJ, Vangen B, Toorawa R, Maldonado-Lutomirsky M, Broedl UC. Empagliflozin as Add-on Therapy in Patients With Type 2 Diabetes Inadequately Controlled With Linagliptin and Metformin: A 24-Week Randomized, Double-Blind, Parallel-Group Trial. Diabetes Care. 2017 Feb;40(2):201-209.

61. Solomon SD, McMurray JJV, Claggett B, de Boer RA, DeMets D, Hernandez AF, Inzucchi SE, Kosiborod MN, Lam CSP, Martinez F, Shah SJ, Desai AS, Jhund PS, Belohlavek J, Chiang CE, Borleffs CJW, Comin-Colet J, Dobreanu D, Drozdz J, Fang JC, Alcocer-Gamba MA, Al Habeeb W, Han Y, Cabrera Honorio JW, Janssens SP, Katova T, Kitakaze M, Merkely B, O'Meara E, Saraiva JFK, Tereshchenko SN, Thierer J, Vaduganathan M, Vardeny O, Verma S, Pham VN, Wilderäng U, Zaozerska N, Bachus E, Lindholm D, Petersson M, Langkilde AM; DELIVER Trial Committees and Investigators. Dapagliflozin in Heart Failure with Mildly Reduced or Preserved Ejection Fraction. N Engl J Med. 2022 Sep 22;387(12):1089-1098.

62. Terauchi Y, Utsunomiya K, Yasui A, Seki T, Cheng G, Shiki K, Lee J. Safety and Efficacy of Empagliflozin as Add-On Therapy to GLP-1 Receptor Agonist (Liraglutide) in Japanese Patients with Type 2 Diabetes Mellitus: A Randomised, Double-Blind, Parallel-Group Phase 4 Study. Diabetes Ther. 2019 Jun;10(3):951-963.

63. van Meijel LA, Tack CJ, de Galan BE. Effect of short-term use of dapagliflozin on impaired awareness of hypoglycaemia in people with type 1 diabetes. Diabetes Obes Metab. 2021 Nov;23(11):2582-2589.

64. Veelen A, Andriessen C, Op den Kamp Y, Erazo-Tapia E, de Ligt M, Mevenkamp J, Jörgensen JA, Moonen-Kornips E, Schaart G, Esterline R, Havekes B, Oscarsson J, Schrauwen-Hinderling VB, Phielix E, Schrauwen P. Effects of the sodium-glucose cotransporter 2 inhibitor dapagliflozin on substrate metabolism in prediabetic insulin resistant individuals: A randomized, double-blind crossover trial. Metabolism. 2023 Mar;140:155396.

65. Vilsbøll T, Ekholm E, Johnsson E, Dronamraju N, Jabbour S, Lind M. Dapagliflozin Plus Saxagliptin Add-on Therapy Compared With Insulin in Patients With Type 2 Diabetes Poorly Controlled by Metformin With or Without Sulfonylurea Therapy: A Randomized Clinical Trial. Diabetes Care. 2019 Aug;42(8):1464-1472.

66. Vilsbøll T, Ekholm E, Johnsson E, Garcia-Sanchez R, Dronamraju N, Jabbour SA, Lind M. Efficacy and safety of dapagliflozin plus saxagliptin versus insulin glargine over 52 weeks as add-on to metformin with or without sulphonylurea in patients with type 2 diabetes: A randomized, parallel-design, open-label, Phase 3 trial. Diabetes Obes Metab. 2020 Jun;22(6):957-968.

67. Watada H, Shiramoto M, Ueda S, Tang W, Asano M, Thorén F, Kim H, Yajima T, Boulton DW, Araki E. Pharmacokinetics and pharmacodynamics of dapagliflozin in combination with insulin in Japanese patients with type 1 diabetes. Diabetes Obes Metab. 2019 Apr;21(4):876-882.

68. Weng J, Zeng L, Zhang Y, Qu S, Wang X, Li P, Fu L, Ma B, Ye S, Sun J, Lu W, Liu Z, Chen D, Cheng Z, Liu H, Zhang T, Zou J. Henagliflozin as add-on therapy to metformin in patients with type 2 diabetes inadequately controlled with metformin: A multicentre, randomized, double-blind, placebo-controlled, phase 3 trial. Diabetes Obes Metab. 2021 Aug;23(8):1754-1764.

69. Wiviott SD, Raz I, Bonaca MP, Mosenzon O, Kato ET, Cahn A, Silverman MG, Zelniker TA, Kuder JF, Murphy SA, Bhatt DL, Leiter LA, McGuire DK, Wilding JPH, Ruff CT, Gause-Nilsson IAM, Fredriksson M, Johansson PA, Langkilde AM, Sabatine MS; DECLARE–TIMI 58 Investigators. Dapagliflozin and Cardiovascular Outcomes in Type 2 Diabetes. N Engl J Med. 2019 Jan 24;380(4):347-357.

70. Cahn A, Raz I, Bonaca M, Mosenzon O, Murphy SA, Yanuv I, Rozenberg A, Wilding JPH, Bhatt DL, McGuire DK, Gause-Nilsson IAM, Fredriksson M, Johansson PA, Jermendy G, Hadjadj S, Langkilde AM, Sabatine MS, Wiviott SD, Leiter LA. Safety of dapagliflozin in a broad population of patients with type 2 diabetes: Analyses from the DECLARE-TIMI 58 study. Diabetes Obes Metab. 2020 Aug;22(8):1357-1368.

71. The EMPA-KIDNEY Collaborative Group; Herrington WG, Staplin N, Wanner C, Green JB, Hauske SJ, Emberson JR, Preiss D, Judge P, Mayne KJ, Ng SYA, Sammons E, Zhu D, Hill M, Stevens W, Wallendszus K, Brenner S, Cheung AK, Liu ZH, Li J, Hooi LS, Liu W, Kadowaki T, Nangaku M, Levin A, Cherney D, Maggioni AP, Pontremoli R, Deo R, Goto S, Rossello X, Tuttle KR, Steubl D, Petrini M, Massey D, Eilbracht J, Brueckmann M, Landray MJ, Baigent C, Haynes R. Empagliflozin in Patients with Chronic Kidney Disease. N Engl J Med. 2023 Jan 12;388(2):117-127.

72. Rosenstock J, Frias J, Páll D, Charbonnel B, Pascu R, Saur D, Darekar A, Huyck S, Shi H, Lauring B, Terra SG. Effect of ertugliflozin on glucose control, body weight, blood pressure and bone

density in type 2 diabetes mellitus inadequately controlled on metformin monotherapy (VERTIS MET). Diabetes Obes Metab. 2018 Mar;20(3):520-529.

73. Gallo S, Charbonnel B, Goldman A, Shi H, Huyck S, Darekar A, Lauring B, Terra SG. Long-term efficacy and safety of ertugliflozin in patients with type 2 diabetes mellitus inadequately controlled with metformin monotherapy: 104-week VERTIS MET trial. Diabetes Obes Metab. 2019 Apr;21(4):1027-1036.

Electronic Supplementary Figure S1. Forest plot for the sub-group analysis based on indications.

Studies	Estimate (95% C.I.)	Ev/Trt	Ev/Ctrl
Anker 2021a	1.000 (0.020, 50.396)	0/2790	0/2789
Charaya 2022	1.040 (0.020, 53.397)	0/50	0/52
Cherney 2020	0.981 (0.019, 50.376)	0/53	0/52
Damman 2020	0.317 (0.013, 8.017)	0/40	1/39
Greeviroj 2023	1.000 (0.019, 53.457)	0/16	0/16
Hollander 2017	1.000 (0.020, 50.692)	0/167	0/167
Lewinski 2022	1.008 (0.020, 51.030)	0/237	0/239
McMurray 2019	7.009 (0.362, 135.763)	3/2368	0/2368
Reis 2022	1.000 (0.019, 52.849)	0/20	0/20
Solomon 2022	5.005 (0.240, 104.289)	2/3126	0/3127
EMPA-KIDNEY 2023	5.008 (0.585, 42.885)	5/3304	1/3305
Heerspink 2020	0.200 (0.010, 4.164)	0/2149	2/2149
Kosiborod 2019	5.041 (0.242, 105.215)	2/613	0/616
Nassif 2021	1.000 (0.020, 50.701)	0/162	0/162
Nassif 2019	1.008 (0.020, 51.158)	0/131	0/132
Nassif 2021c	0.970 (0.019, 50.361)	0/33	0/32
Spertus 2022	1.000 (0.020, 50.615)	0/226	0/226
Antlanger 2022b	1.000 (0.018, 54.830)	0/11	0/11
Veelen 2023	1.000 (0.019, 53.890)	0/14	0/14
**Subgroup 3 (I^2=0 % , P=0.993)**	**1.494 (0.675, 3.304)**	**12/15510**	**4/15516**
Baker 2019	1.048 (0.042, 26.297)	1/105	0/36
Bode 2021	0.318 (0.013, 8.029)	0/43	1/42
Buse 2018	10.574 (1.411, 79.222)	20/525	1/268
Danne 2018	4.470 (0.240, 83.336)	4/524	0/258
Garcia-Tirado 2022	3.000 (0.114, 78.814)	1/18	0/17
Haidar 2022	1.000 (0.019, 52.217)	0/27	0/27
Henry 2015	18.908 (1.127, 317.239)	17/234	0/117
Henry 2015b	0.717 (0.028, 18.584)	1/57	0/13
Kuhadiya 2016	3.065 (0.132, 70.939)	2/17	0/9
Mathieu 2018 & Mathieu 2020	10.403 (1.389, 77.931)	20/541	1/272
Sands 2015	6.034 (0.268, 135.993)	2/16	0/17
Van Meijel 2020	1.000 (0.019, 53.659)	0/15	0/15
Watada 2018	0.509 (0.010, 26.973)	0/28	0/14
**Subgroup 1 (I^2=0 % , P=0.698)**	**3.577 (1.586, 8.070)**	**68/2150**	**3/1105**
Bhatt 2021	0.503 (0.092, 2.758)	2/605	4/611
Bhatt 2021b	2.147 (1.137, 4.054)	30/5291	14/5286
Brown 2020	1.062 (0.020, 55.082)	0/32	0/34
Wiviott 2019 & Cahn 2020	2.253 (1.140, 4.449)	27/8574	12/8569
Cannon 2020	4.760 (1.108, 20.452)	19/5493	2/2745
Carbone 2020	1.114 (0.021, 59.200)	0/17	0/19
Cherney 2017a	0.983 (0.019, 49.672)	0/349	0/343
Cherney 2017b	0.996 (0.020, 50.298)	0/518	0/516
Cherney 2017c	1.094 (0.021, 56.367)	0/42	0/46
Cherney 2021	0.507 (0.010, 25.742)	0/184	0/93
Cherney 2023	0.494 (0.010, 24.958)	0/527	0/260
Curovic 2022	0.886 (0.017, 47.358)	0/17	0/15
de Boer 2020	1.098 (0.021, 57.072)	0/30	0/33
de Boer 2020-2	0.536 (0.010, 27.625)	0/62	0/33
Fioretto 2018	1.006 (0.020, 51.020)	0/160	0/161
Rosenstock 2018b & Gallo 2019	1.527 (0.062, 37.655)	1/412	0/209
Griffin 2020	1.000 (0.019, 52.849)	0/20	0/20
Halvorsen 2023	3.038 (0.123, 75.144)	1/158	0/159
Ikeda 2015	0.202 (0.004, 10.293)	0/328	0/66
Ito 2017	1.062 (0.020, 55.082)	0/32	0/34
Ito 2021	1.783 (0.033, 95.963)	0/11	0/20
Ji 2023	0.502 (0.010, 25.538)	0/146	0/73
Kadowaki 2017	0.972 (0.019, 49.661)	0/70	0/68
Katsiadas 2022	0.965 (0.019, 49.477)	0/56	0/54
Kawamori 2018	0.512 (0.010, 26.025)	0/182	0/93
Laffel 2023	0.663 (0.027, 16.568)	0/52	1/105
Nishimura 2015	0.544 (0.010, 28.409)	0/39	0/21
Perkovic 2019	11.035 (1.423, 85.546)	11/2200	1/2197
Pollock 2019	1.503 (0.061, 37.107)	1/297	0/148
Rau 2021	1.098 (0.021, 57.884)	0/20	0/22
Ridderstrale 2014 & Ridderstrale 2018	1.020 (0.020, 51.450)	0/765	0/780
Sargeant 2022	1.000 (0.019, 51.844)	0/34	0/34
Satirapoj 2019	1.035 (0.020, 53.951)	0/28	0/29
Shimada 2018	0.307 (0.006, 16.334)	0/37	0/11
Softeland 2017	0.497 (0.010, 25.195)	0/222	0/110
Vilsboll 2019 & Vilsboll 2020	0.327 (0.013, 8.061)	0/324	1/319
Vilsboll 2019 & Vilsboll 2020-2	0.327 (0.013, 8.061)	0/324	1/319
Weng 2021	0.499 (0.010, 25.273)	0/327	0/163
Yabe 2023	0.985 (0.019, 50.380)	0/65	0/64
Antlanger 2022a	1.087 (0.020, 59.399)	0/11	0/12
Jurgens 2021	1.000 (0.019, 51.491)	0/45	0/45
Kashyap 2020	0.478 (0.008, 27.437)	0/11	0/5
Kuchay 2023	1.000 (0.020, 50.791)	0/125	0/125
Op den Kamp 2021	1.000 (0.019, 52.443)	0/24	0/24
**Subgroup 2 (I^2=0 % , P=1.000)**	**1.638 (1.160, 2.313)**	**92/28266**	**36/24093**
**Overall (I^2=0 % , P=1.000)**	**1.792 (1.334, 2.407)**	**172/45926**	**43/40714**

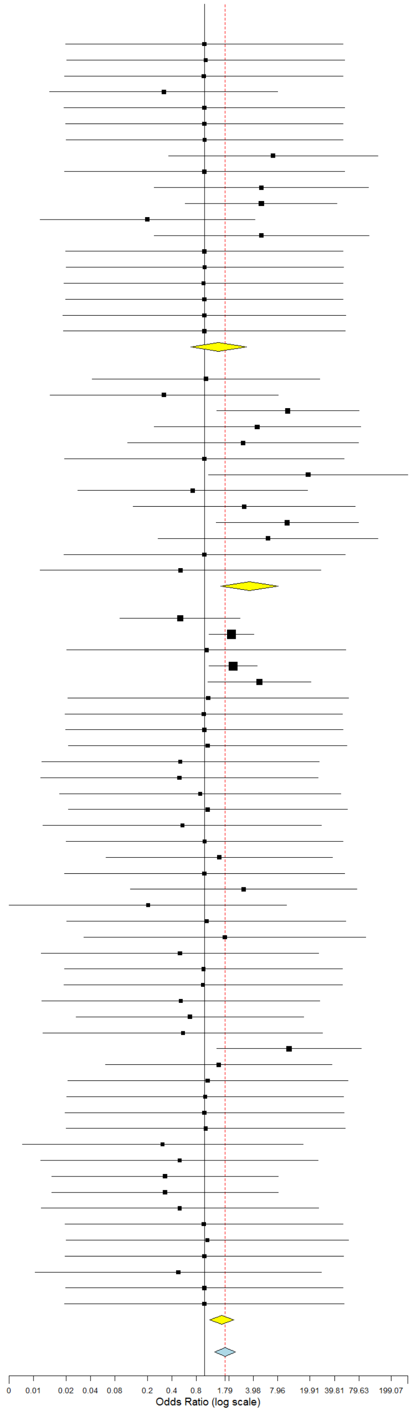

Subgroup 1 indicates T1D; subgroup 2 indicates T2D; and subgroup 3 represent non-diabetic indications.

Electronic Supplementary Figure S2. Funnel plot for the risk of DKA with SGLTi.

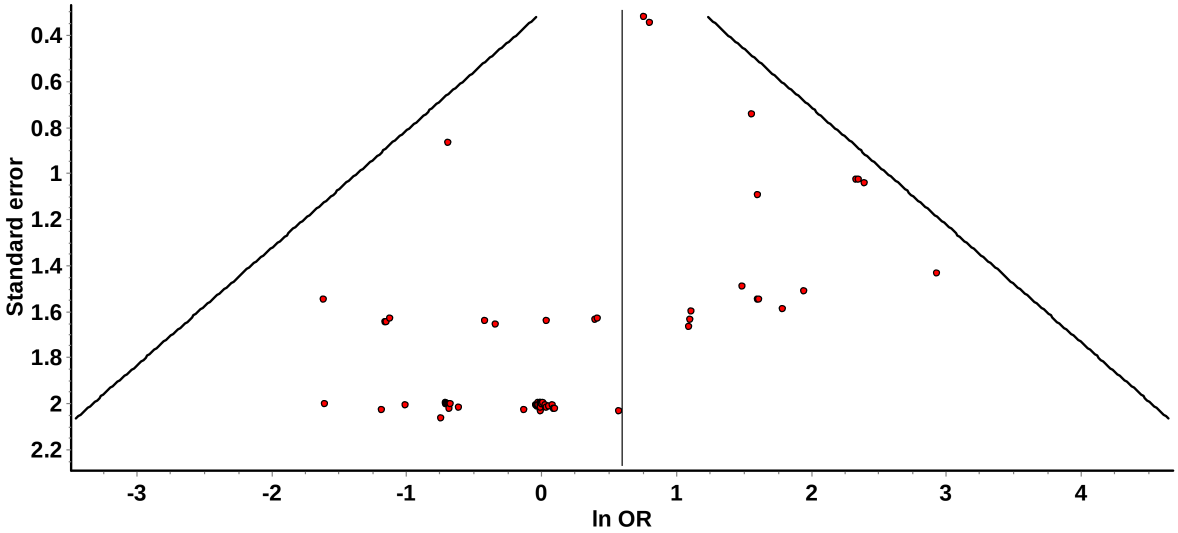

Electronic Supplementary Figure S3. Funnel plot for the risk of DKA with dapagliflozin.

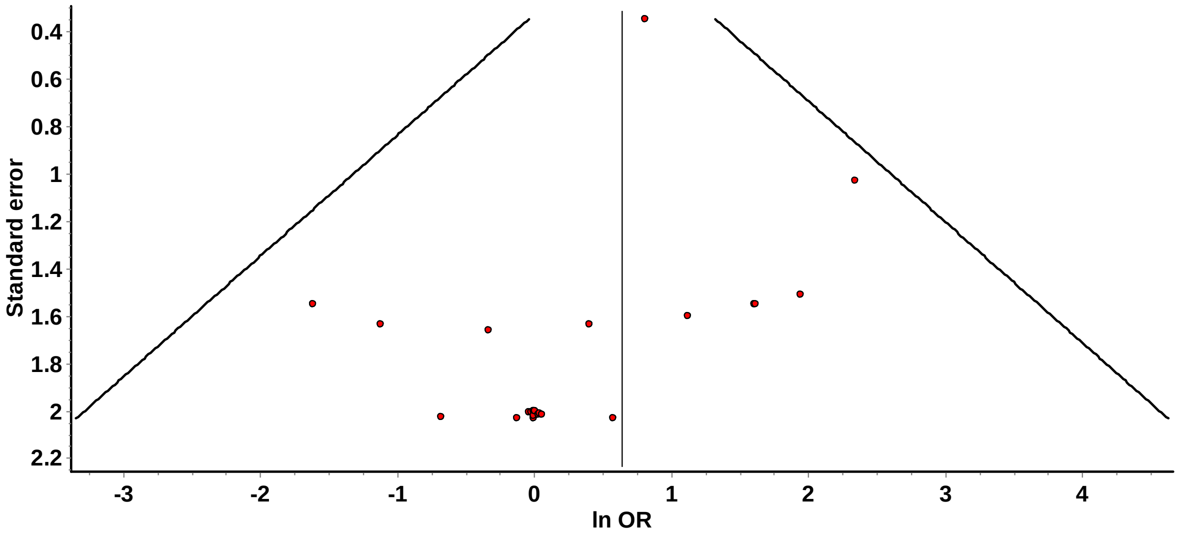

Electronic Supplementary Figure S4. Funnel plot for the risk of DKA with sotagliflozin.

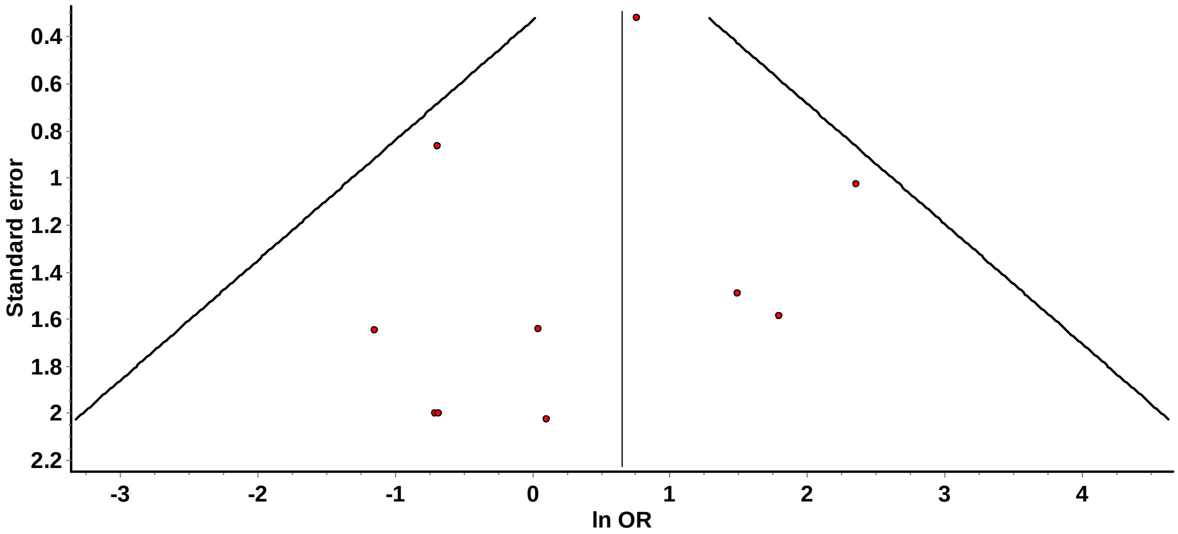

Electronic Supplementary Figure S5. Funnel plot for the risk of DKA with canagliflozin.

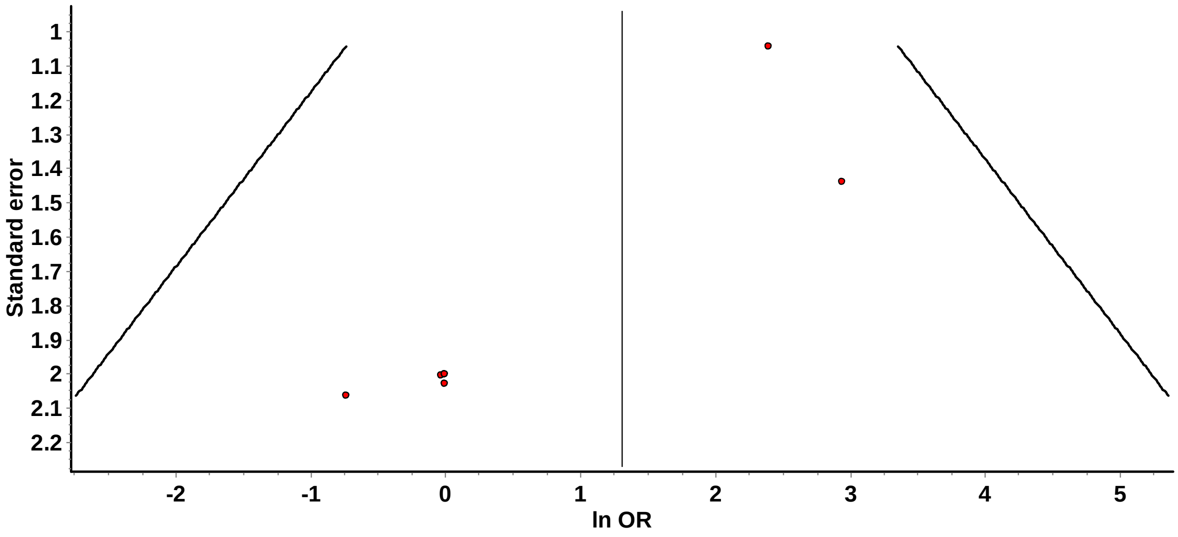

Electronic Supplementary Figure S6. Funnel plot for the risk of DKA with low doses of SGLT2i.

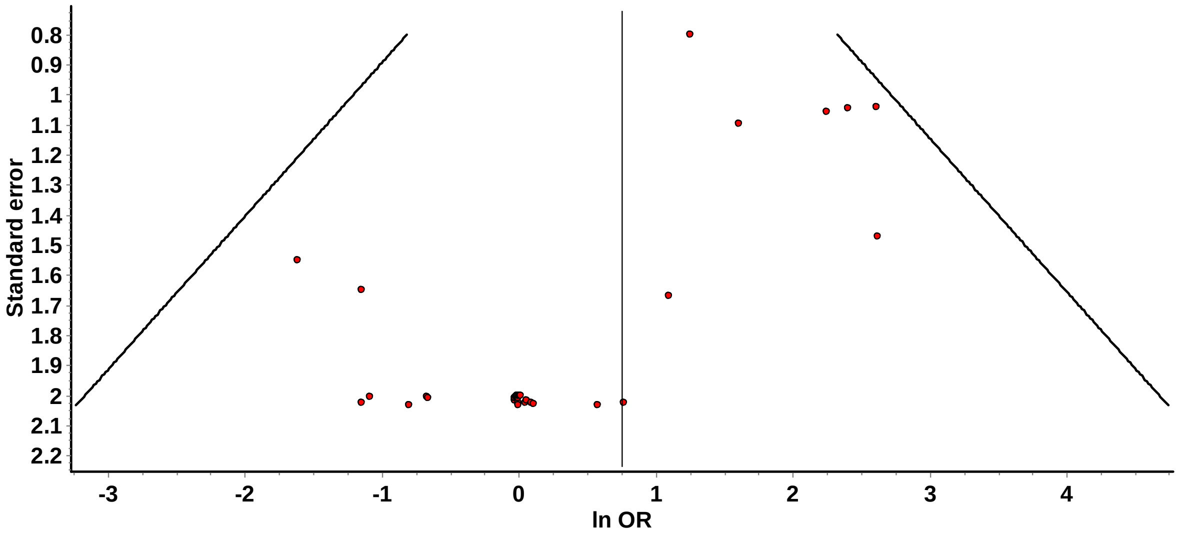

Electronic Supplementary Figure S7. Funnel plot for the risk of DKA with high doses of SGLT2i.

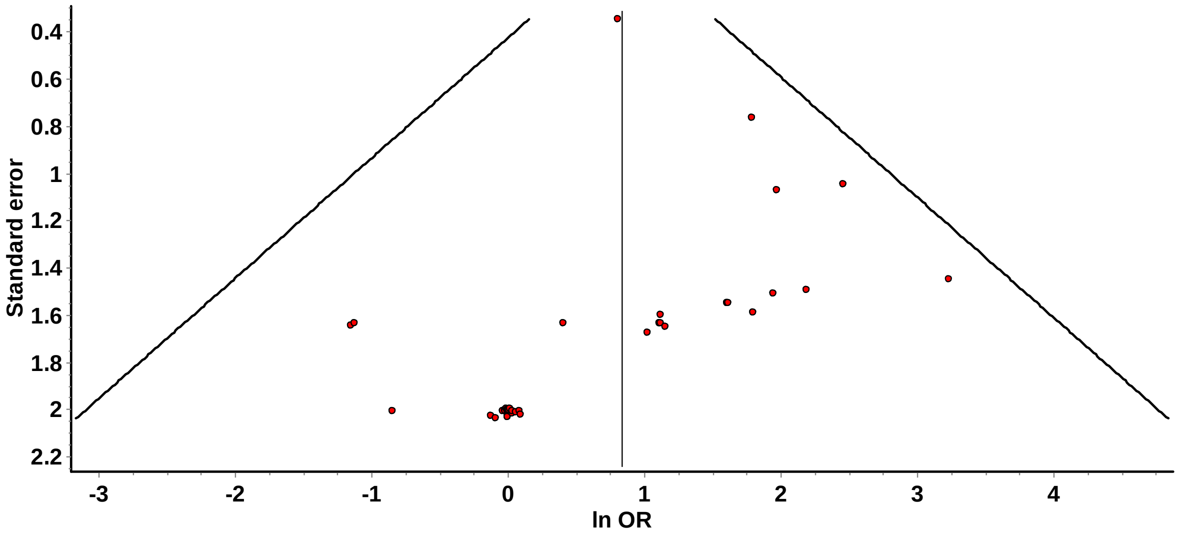

Electronic Supplementary Figure S8. Leave-one-out sensitivity analysis for the risk of DKA.

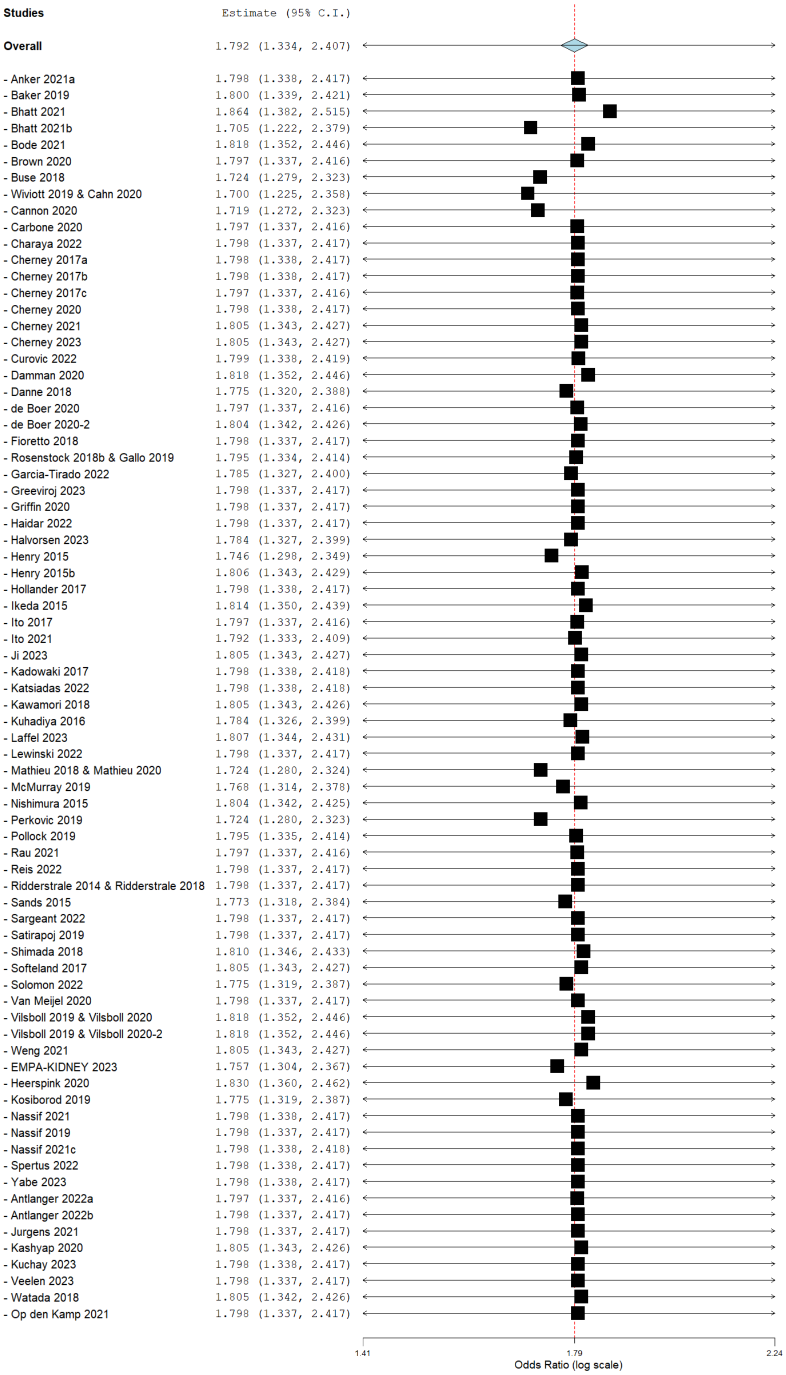

The black square boxes indicate the pooled estimates with the removal of data from the respective studies.
